# A Comprehensive Analysis of Calmodulin-Like Proteins of *Glycine max* Indicates Their Role in Calcium Signaling and Plant Defense Against Insect Attack

**DOI:** 10.3389/fpls.2022.817950

**Published:** 2022-03-09

**Authors:** Manisha Yadav, Jyotsna Pandey, Amrita Chakraborty, Md. Imtaiyaz Hassan, Jiban Kumar Kundu, Amit Roy, Indrakant Kumar Singh, Archana Singh

**Affiliations:** ^1^Department of Botany, Hansraj College, University of Delhi, New Delhi, India; ^2^EVA4.0 Unit, Faculty of Forestry and Wood Sciences, Czech University of Life Sciences Prague, Prague, Czechia; ^3^Centre for Interdisciplinary Research in Basic Sciences, Jamia Millia Islamia, New Delhi, India; ^4^Plant Virus and Vector Interactions Group, Crop Research Institute, Prague, Czechia; ^5^Molecular Biology Research Laboratory, Department of Zoology, Deshbandhu College, University of Delhi, New Delhi, India; ^6^DBC-i4 Center, Deshbandhu College, University of Delhi, New Delhi, India

**Keywords:** Calmodulin like proteins (CMLs), soybean, *Spodoptera litura*, calcium signaling, signaling compounds, wounding, plant-insect interaction

## Abstract

The calcium (Ca^2+^) signaling is a crucial event during plant-herbivore interaction, which involves a transient change in cytosolic Ca^2+^ concentration, which is sensed by Ca^2+^-sensors, and the received message is transduced to downstream target proteins leading to appropriate defense response. Calmodulin-like proteins (*CMLs*) are calcium-sensing plant-specific proteins. Although *CMLs* have been identified in a few plants, they remained uncharacterized in leguminous crop plants. Therefore, a wide-range analysis of *CMLs* of soybean was performed, which identified 41 true CMLs with greater than 50% similarity with *Arabidopsis CMLs*. The phylogenetic study revealed their evolutionary relatedness with known *CMLs*. Further, the identification of conserved motifs, gene structure analysis, and identification of *cis*-acting elements strongly supported their identity as members of this family and their involvement in stress responses. Only a few *Glycine max CMLs (GmCMLs)* exhibited differential expression in different tissue types, and rest of them had minimal expression. Additionally, differential expression patterns of *GmCMLs* were observed during *Spodoptera litura*-feeding, wounding, and signaling compound treatments, indicating their role in plant defense. The three-dimensional structure prediction, identification of interactive domains, and docking with Ca^2+^ ions of *S. litura*-inducible GmCMLs, indicated their identity as calcium sensors. This study on the characterization of *GmCMLs* provided insights into their roles in calcium signaling and plant defense during herbivory.

## Introduction

Plants being sessile are constantly attacked by various environmental cues that can cause abiotic or biotic stresses (Stotz et al., 2000). Among all, insect attack is crucial biotic stress, affecting the plants’ fitness, performance, and productivity (Stotz et al., 2000; Conrath et al., 2002; Dodd et al., 2010; Hancock et al., 2015; Singh et al., 2018, 2020a,b,2021c; Chen and Mao, 2020; Keshan et al., 2021; Singh and Singh, 2021). To protect themselves, plants activate their defense mechanism and produce different defensive compounds like secondary metabolites, volatile organic compounds, protease inhibitors, and other antiherbivore chemicals (Gatehouse, 2002; Halitschke and Baldwin, 2004; Kliebenstein, 2004; Schuler, 2011; Vadassery et al., 2012b; Lortzing and Steppuhn, 2016; Singh et al., 2021a,c). This activation of biosynthesis of defensive compounds is governed by certain early events that facilitate the detection of herbivory and signal transduction. However, stimulus perception and signal transduction during herbivory is not understood well. Moreover, plants must discriminate among various environmental stimuli to trigger a specific response by activating downstream cellular signaling. Therefore, it is mandatory to determine the perception molecules, crucial players of signal transduction, and molecular mechanism behind plant defense against herbivory (Kong et al., 2013; Singh et al., 2016, 2020c; Kumar et al., 2020).

Plants recognize herbivore attack by wounding patterns and interaction of herbivore/damage-associated molecular patterns (HAMPs/DAMPs) with pattern recognition receptors (PRRs) (Mithöfer and Boland, 2008). This interaction activates appropriate downstream signal transduction pathways for achieving stimulus-specific responses. The process of coupling the perception of feeding herbivores to plant adaptive response involves transient changes in the intracellular Ca^2+^ concentration (Maffei et al., 2004; Thor and Peiter, 2014). During the process, an increase in Ca^2+^ levels occurs due to the transport of Ca^2+^ across the membrane or from subcellular organelles, which is facilitated by plasma membrane Ca^2+^ -permeable channels, such as cyclic nucleotide-gated channels (CNGCs), glutamate receptor-type cation channel (GLRs), stretch-activated Ca^2+^ channel (OSCAs), and MID1-complementing activity (MCA) families and vacuolar channel TWO-PORE CHANNEL1 (TPC1) (Maffei et al., 2004; Dodd et al., 2010; Vadassery et al., 2012a; Kiep et al., 2015; DeFalco et al., 2016; Vincent et al., 2017; Meena et al., 2019; DeFalco and Zipfel, 2021). This transport of Ca^2+^ causes spatial and temporal variations in cellular distribution, frequency, amplitude, kinetics of Ca^2+^, and intracellular Ca^2+^ level leading to the generation of calcium signature, which encrypts information from primary stimuli to encode specific intracellular responses (Ranty et al., 2016). These calcium signals are decoded further by various calcium sensor proteins that consist of canonical Ca^2+^ binding EF-hand motif with conserved helix-loop-helix structure coordinating one Ca^2+^ (Lecourieux et al., 2002; Gifford et al., 2007). These proteins perceive and interpret Ca^2+^ signals through binding to Ca^2+^ ions leading to a change in their conformation and functions (Aldon et al., 2018). In plants, Ca^2+^ binding proteins are represented by complex families. They can be either Ca^2+^ responders, which convey the signal *via* enzymatic reactions, such as Ca^2+^-dependent protein kinases (CDPKs), or the non-catalytic sensor relay proteins, such as Calmodulins (CaMs), Calmodulin-like proteins (CMLs), and calcineurin B-like proteins (CBLs), which are activated upon binding to Ca^2+^, which promote downstream signaling (DeFalco et al., 2010).

Calmodulin like proteins are principal representative of Ca^2+^ sensor proteins (DeFalco et al., 2010; Kumar et al., 2020) and are characterized by two to six EF-hand motifs and are known to evolve from ancestor CaM, sharing at least 15% amino acid identity (Perochon et al., 2011; Bender and Snedden, 2013). So far, CMLs are pointed out to be involved in the developmental process, plant immunity, and stress responses by targeting many kinases, ion transporters, metabolic enzymes, phosphatases, and transcription factors (Reddy et al., 2011). The EF-hand motifs show a high affinity toward cooperative binding with Ca^2+^ that expose the hydrophobic surface, which allows them to interact with downstream target proteins (Vadassery et al., 2012a). *CML*8, *CML*37, *CML*38, and *CML*39 are responsive to drought, salinity, and hormonal treatment in *Arabidopsis* (Vanderbeld and Snedden, 2007; Park et al., 2010). *CML*9 is regulated by abscisic acid (ABA) and *Pseudomonas syringae* infection (Magnan et al., 2008; Leba et al., 2012). *CML*42 is known to cause aberration in trichomes (Dobney et al., 2009), and *CML*24 is involved in alterations in flowering time and ion stress (Delk et al., 2005; Hubbard et al., 2008). Plants possess a repertoire of *CML*s, which are unique to them with 50 members in *Arabidopsis* (McCormack et al., 2005), 52 in *Solanum lycopersicum* (Munir et al., 2016), 19 in *Lotus japonicas* (Liao et al., 2017), and 62 in *Vitis vinifera* (Vandelle et al., 2018). Few *CMLs* of *Arabidopsis thaliana* (*CML9, 11, 12, 16, 17, 23*, and *42*) are upregulated upon employing oral secretion of *Spodoptera littoralis* (Vadassery et al., 2012a,b). *CML37* and *CML42* act antagonistically to regulate stress responses in *Arabidopsis* by altering phytohormone signals (Heyer et al., 2021). However, the involvement of these Ca^2+^ sensors has not been investigated much in crop plants. To achieve more insights into the function of *CMLs* and recognize their involvement in plant defense against herbivory, it would be crucial to investigate them during soybean-*Spodoptera litura* interaction.

*Glycine max*, commonly known as soybean, is a dietary staple crop of Asian countries and a native legume plant of Southeastern Asia (Soyastats, 2010; Badole and Bodhankar, 2012; Badole and Mahamuni, 2013; Naresh et al., 2019). It consists of a balanced proportion of amino acids essential for the growth of the human body (Naresh et al., 2019). The genome of soybean is fully sequenced, and the processes of gene regulation, disease resistance, and nodulation are well defined (Gresshoff and Ferguson, 2017). However, insect pests can adversely affect the yield and quality of soybean (Cui et al., 1997). *S. litura* (common cutworm), a polyphagous insect, is one of the major destructive and widespread soybean pests throughout the Asia-Pacific region. Plants have employed various constitutive and induced defense strategies along with sophisticated signaling networks to combat predation by herbivores (Singh et al., 2021b). The induced responses are more effective for plants and lead to increased tolerance against herbivores (Stotz et al., 2000; Zhang and Yang, 2000; Fan et al., 2012). *S. litura*, a nocturnal moth and a serious polyphagous defoliator, infests soybean plants through the vigorous eating pattern of larvae (Singh et al., 2021b). The proteolytic activity of the gut serine protease of the larvae is mainly responsible for the significant damage to crops (Vasudev and Sohal, 2016). Therefore, it is essential to elucidate how *S. litura*-infestation induces defense signaling in *G. max* evoking the plant defense mechanism.

In the present study, a comprehensive investigation of *CMLs* of *G. max* was executed across the genome. We studied the genomic organization, evolutionary and phylogenetic relationships, gene structure analysis, and identification of EF-hand motifs of *Glycine max CMLs (GmCMLs)*. We also evaluated the mRNA levels of *GmCMLs* in different tissue types. The investigation on change in mRNA levels of *GmCMLs* during *S. litura*-infestation revealed the involvement of 36 *GmCMLs* in plant defense against herbivory. We also screened the promoter sequences of *GmCMLs* to identify *cis*-acting elements that revealed the presence of Jasmonic acid (JA)-response elements and salicylic acid (SA)-related elements. Further, transcript profiling of *S. litura*-inducible *CMLs* upon defense-signaling compound treatments was also executed, which revealed their differential expression. We also predicted the three-dimensional structure of *S. litura*-inducible GmCMLs and its interaction with Ca^2+^, which confirmed their identity and involvement in Ca^2+^ signaling. This analysis has offered promising candidates that can function as an essential component of Ca^2+^ signaling during *G. max*-*S. litura* interaction and can be valuable for deciphering the early events involved in the upregulation of plant defense in soybean and beyond.

## Materials and Methods

### Sequence Retrieval, Phylogenetic Analysis, and Similarity Search of Calmodulin-Like Proteins of *Glycine max*

The protein and gene sequences of *CMLs* of *G. max* were identified and retrieved from the soybean database^1^ and phytozome database^2^. The DNA and peptide sequences of GmCMLs were evaluated manually utilizing BLASTN and BLASTP^3^. The base pair lengths and number of amino acids for each *GmCMLs* were retrieved from NCBI^4^.

To study the evolutionary relationships among model plants, monocot, and leguminous dicot, amino acid sequences of homologous GmCMLs from *Medicago truncatula*, *Arabidopsis thaliana*, and *Oryza sativa* were retrieved from Phytozome (see text footnote 2), TAIR^5^, and Oryzabase^6^, respectively. The amino acid sequences were then submitted to multiple sequence alignment using Clustal Omega^7^ (Madeira et al., 2019). The generated multiple sequence alignment was subjected to “construct” phylogenetic tree. The details of constructed phylogenetic tree were downloaded using “Download” phylogenetic data and saved in newick format. For better presentation of the phylogenetic tree, the saved phylogenetic tree was uploaded on iTOL^8^ (Letunic and Bork, 2021).

The identity of GmCML peptide sequences was compared with the protein sequence of CMLs of *Arabidopsis thaliana* using the BLAST engine of the TAIR database^9^.

### Expression Analysis of *Glycine max CMLs* During *Spodoptera litura*-Infestation

#### Plant Growth

The soybean seeds (Pusa 9712) were washed, soaked for 2 h, and kept at 26−27°C in a dark chamber between 2 layers of tissue paper, with continuous moistening with distilled water for germination. After 3–4 days, the germinated seedlings were transferred to the sterilized soilrite containing pots. The seedlings were grown in a plant growth chamber under controlled conditions (photoperiod: 16 h light and 8 h dark; temperature: 26−27°C; humidity: 55–60%) and watered regularly.

#### Rearing and Maintenance of *Spodoptera litura*

The larvae of *S. litura* were procured from ICAR – National Bureau Of Agricultural Insect Resources, Bangalore, India, and reared in the laboratory using standard protocols at 27°C and 65–70% relative humidity on a 14/10 h light/dark cycle (Singh et al., 2021b). The freshly molted fourth instar larvae were starved for 12 h before releasing them on plants for experimentation and bioassays.

#### *Spodoptera litura*-Infestation and Mechanical Wounding on Soybean Plants and Sample Collection

For *S. litura*-infestation, one-month-old soybean plants were exposed to one larva per plant. Plants without any treatments were served as control. Mechanical wounding was performed according to Singh et al. (2008). The entire shoot of treated (sample) and untreated (control) plants, after 1 h of infestation/wounding from three independent experiments, was harvested and immediately snap-frozen at −80°C for further analysis.

#### RNA Isolation and cDNA Synthesis

Trizol (TRI) reagent (Sigma Aldrich, Spruce Street, St. Louis, MO, United States) was used to isolate RNA as per the given protocol (Singh et al., 2018, 2021b; Keshan et al., 2021). The RNA concentrations were measured on a spectrophotometer, and RNA integrity was checked by agarose gel electrophoresis. The isolated RNA was used to synthesize first-strand cDNA using an iScript cDNA synthesis kit (Bio-Rad, Hercules, CA, United States).

#### Primer Design and Gene Expression Analysis Using qPCR

Primer 3 (v.0.4.0)^10^ tool was used to design real-time (RT) PCR primers. Applied Biosystems™ 7500 RT PCR System (Thermo Fisher Scientific, Waltham, MA, United States) was used to perform RT-qPCR. Each 10 μl reaction mix contained 1 μl of cDNA samples, 0.5 μl of 10 μM gene-specific primers, and 5 μl of SYBR green supermix (Bio-Rad, Hercules, CA, United States). The soybean elongation factor gene was used as an internal control. The qPCR data were analyzed utilizing the 2^–ΔΔCT^ method. The 2^–ΔΔCT^ values obtained by qPCR were transformed to log^2^ to lessen noise level. For statistical analysis, ANOVA and Tukey’s test (*P* < 0.05) were applied and a criterion of greater than two-fold induction/reduction level was taken into consideration to select differentially expressing genes according to a previous report (Singh et al., 2008). The heat map was generated by Graphpad Prism Software^11^ using qPCR data.

### Expression Analysis of *Glycine max CMLs* Upon Treatment With Signaling Compounds

For treatment with signaling compounds, an equal volume of 100 μM of methyl-jasmonate, 50 μM ethephon, 5 μM salicylic acid, and only water/0.5% (v/v) ethanol in water (control) were sprayed on healthy soybean plants according to the previous studies (Stotz et al., 2000; Singh et al., 2008). The plants were maintained under the same conditions but in individual enclosures. The leaves of treated (sample) and untreated (control) plants were harvested after 1 h of treatment in biological triplicates and immediately snap-frozen at −80°C for further analysis. For gene expression analysis, qPCR for 36 *GmCML*s (differentially expressed on *S. litura*-infestation) was performed, and the data were analyzed using the 2^–ΔΔCT^ method. For statistical analysis, ANOVA and Tukey’s test (*P* < 0.05) were applied and a criterion of greater than two-fold induction/reduction level was taken into consideration to select differentially expressing genes. The heat map was generated by Graphpad Prism Software (see text footnote 11) using qPCR data.

### *In silico* Analysis of True *Glycine max CMLs*

The subcellular localization of different GmCMLs was predicted using WoLF PSORT^12^. The molecular weight and isoelectric point of each GmCMLs were computed using the “Compute pI/MW tool” of ExPASy^13^. The presence of EF-hands was checked using the InterPro database^14^. The MEME software^15^ was used to detect and generate logo plots of the EF-hands motifs.

### Exon-Intron Determination

The organization of exons and introns in *GmCMLs* was deciphered using Gene Structure Display server GSDS2.0^16^ (Hu et al., 2015).

### Tissue-Specific Gene Expression Profiling

The expression profile values [Reads/Kb/Million (RPKM) normalized data] for different tissues, that is, flower, young leaf, green pods, root, nodule, stem, and seed, were retrieved from SoyBase Expression Explorer^17^ (Waese et al., 2017). The heat map was illustrated using RPKM values for tissue-specific expression profiling of *GmCMLs* on Graphpad Prism Software (see text footnote 11).

### Identification of microRNA (miRNA) Targets of *Glycine max CMLs*

Plant small-RNA target (psRNATarget) analysis server was used to predict miRNA target sites in *GmCMLs* transcripts with default parameters. *G. max*-specific mature miRNA sequences were downloaded from the miRNA database (miRBase)^18^ (Bhatia et al., 2019).

### Interaction of *Glycine max* CMLs With Other Proteins

To deduce the direct and indirect interaction of GmCMLs with other proteins, the sequences of all GmCMLs were subjected to the String database^19^ (Szklarczyk et al., 2021). The GmCML protein sequences were submitted as multiple proteins with organisms selected as *G. max*. The submission of sequences redirected to a new window, where proteins matching with input sequences along with identity, bitscore, and *e*-value were listed. Protein interaction list was saved by clicking on “Mapping”, and further to find interaction networks proceeded with “continue.”

### Detection of Regulatory Elements in the Promoter Region of *Glycine max CMLs*

To detect the regulatory elements present in the 5′ upstream region of *GmCMLs*, 1 kb of the genomic DNA sequences before the initiation codon (ATG) of each *GmCMLs* were saved from NCBI and subjected to Plant Care analysis^20^ (Lescot et al., 2002).

### Three-Dimensional Structure Prediction of Selected *Glycine max* CMLs by Homology Modeling

The homology modeling of *S. litura*-inducible GmCMLs was conducted to predict their three-dimensional structure. The selected *S. litura*-inducible GmCMLs were used as query sequences for homology modeling and searched against the Protein Data Bank (PDB) to identify the closely related known protein sequence. The data were submitted to the Phyre^2^ Protein Homology/Analogy recognition Engine http://www.sbg.bio.ic.ac.uk/∼phyre2/html/page.cgi?id=index to construct the protein 3-D structure by homology modeling under intensive modeling mode (Kelley et al., 2015). Further, to check the stereochemical quality of protein tertiary structures, the PROCHECK server was used http://saves.mbi.ucla.edu/ (Laskowski et al., 1996).

### Interaction of Selected *Glycine max* CMLs With Ca^2+^

To show the interaction between Ca^2+^ and GmCMLs, the InChI format of Ca^2+^ was derived from PubChem^21^ (Kim et al., 2019). For docking studies, the InChI format was converted to.pdb using OpenBabel--Chemical file format converter^22^ (O’Boyle et al., 2011). The interaction studies were performed for true GmCMLs that were differentially regulated by *S. litura*-infestation using PATCHDOCK https://bioinfo3d.cs.tau.ac.il/PatchDock/php.php (Schneidman-Duhovny et al., 2005). Different parameters used for docking studies were clustering root-mean-square deviation (RMSD) = 4, and the complex type is protein and small ligand. The top ten Patchdock docking results were further refined using FireDock^23^ (Mashiach et al., 2008). The GmCMLs were docked with Ca^2+^ based on the number of EF-hand motifs. The global energy scores of docked molecules were compared with that of the Calmodulin 2 protein of *Arabidopsis thaliana* with Ca^2+^.

## Results

### Genome-Wide Survey, Phylogenetic Study, and Similarity Search Identified True *Glycine max CMLs* in *Glycine max* Genome

**FIGURE 1:**
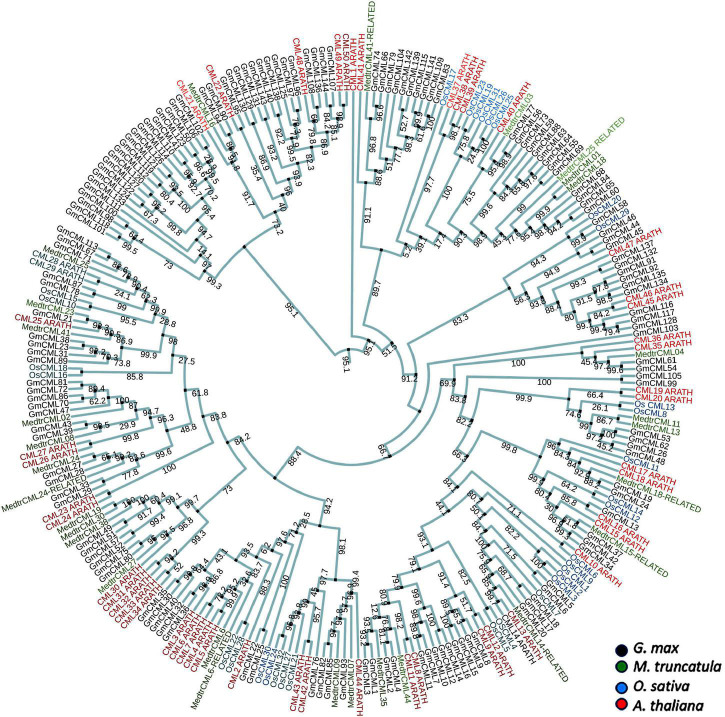
The phylogenetic analysis of CMLs of *Glycine max, Medicago truncatula, Oryza sativa*, and *Arabidopsis thaliana.* The alignment for phylogenetic tree was performed with Clustal Omega using full-length protein sequences. The phylogenetic tree was constructed using iTOL. All the *Glycine max* CMLs (GmCMLs) are presented with black, *Medicago truncatula* CMLs are presented with green, *Oryza sativa* CMLs are presented with blue, *and Arabidopsis thaliana CMLs* are presented with red. The bootstrap values are provided in the mid of the nodes.

A total of 144 CMLs were predicted in the soybean genome by Zeng et al. (2017) in a preliminary study. The protein sequences of these predicted CMLs were procured and named as GmCMLs. Phylogenetic analysis of GmCMLs with CMLs of other plants, including monocots and leguminous dicots was executed, which showed evolutionary relatedness among them. This analysis revealed that GmCML101, 118, 98, and 100 shared ancestry. GmCML113, 167, 71, and CML25 from *M. truncatula* and CML28 and CML29 from *A. thaliana* diverged recently (Figure 1). Further, the GmCMLs were compared with CMLs of *A. thaliana*, revealing that 41 CMLs showed 50% or more sequence similarity with CMLs of *A. thaliana.* Sixteen of the GmCMLs showed higher sequence similarity (85–70%), whereas 25 GmCMLs showed more than 50% sequence similarity with CMLs of *A. thaliana*. The rest of the CMLs (103 GmCMLs) exhibited less than 50% homology, and 3 of them did not match with any of the CMLs of *A. thaliana*. With an identity of 50% as a threshold, 41 GmCMLs with 50% or more identities were labeled as true GmCMLs. A list of GmCMLs and their homologous CML of *A. thaliana* and their percentage similarity is presented in Supplementary Table 1.

### Differential Expression of *Glycine max CMLs* During *Spodoptera litura*- Infestation and Wounding

**FIGURE 2:**
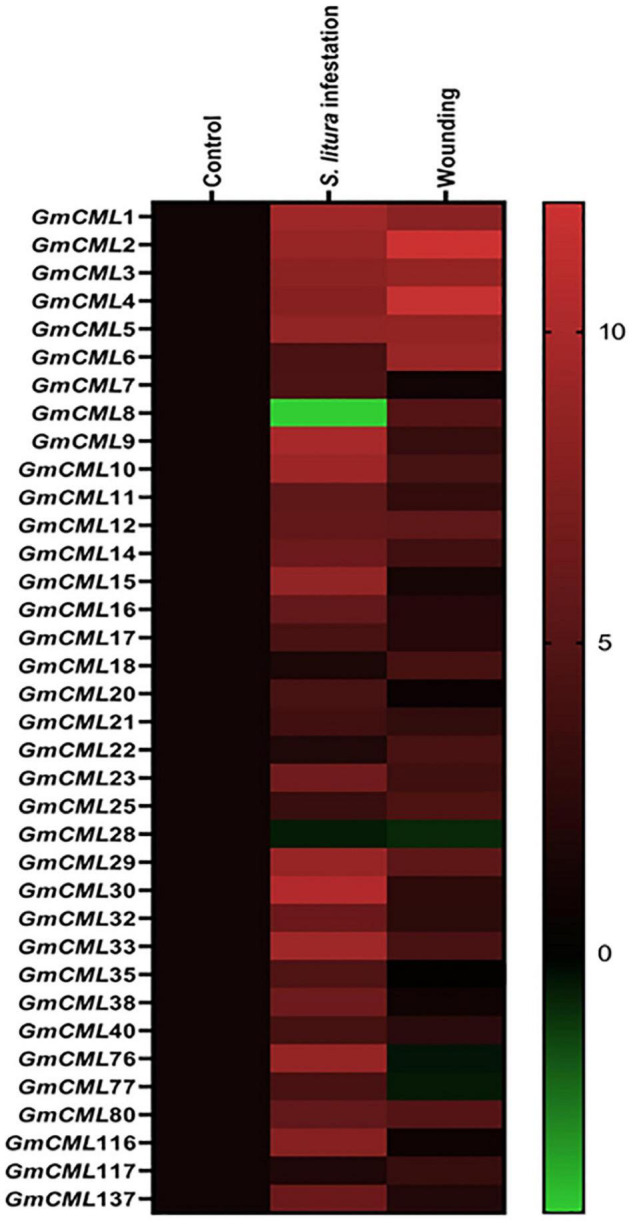
Heat map showing expression profile of *GmCMLs* upon *S. litura*-infestation and wounding. The expression profile of *GmCML*s upon *S. litura*-infestation and wounding is represented as heat map, generated with the help of Graphpad Prism software using log^2^ values of expression data obtained from qPCR. The qPCR data were analyzed utilizing 2^–ΔΔCT^ method and statistical analysis was performed using ANOVA and Tukey’s test (*P* < 0.05).

To gain information on the involvement of *GmCMLs* in plant defense against *S. litura*-infestation, the expressions of true 41 *GmCML*s were checked using qPCR with gene-specific primers (Supplementary Table 2), and the results indicated that 34 of the *GmCMLs* were upregulated while 2 of the *GmCMLs* were downregulated. *GmCML*30 showed a maximum change in expression while *GmCML*33, 9, 10, 1, and *GmCML*2, 76, 29, 3, 15, and 5 also showed a high level of expression (Figure 2; Supplementary Figure 1). The results demonstrated that these *GmCMLs* were responsive to *S. litura*-infestation. However, a different transcript pattern was observed when plants were wounded/mechanically damaged; 24 of the *GmCMLs* were upregulated, and 5 *GmCMLs* were downregulated. Seven of the *GmCMLs* (*GmCMLs* 7, 20, 21, 35, 76, 77, and 116) exhibited a significant change in mRNA expression specifically during *S. litura*-infestation.

### Differential Expression of *Glycine max CMLs* Upon Foliar Application of Signaling Compounds

**FIGURE 3:**
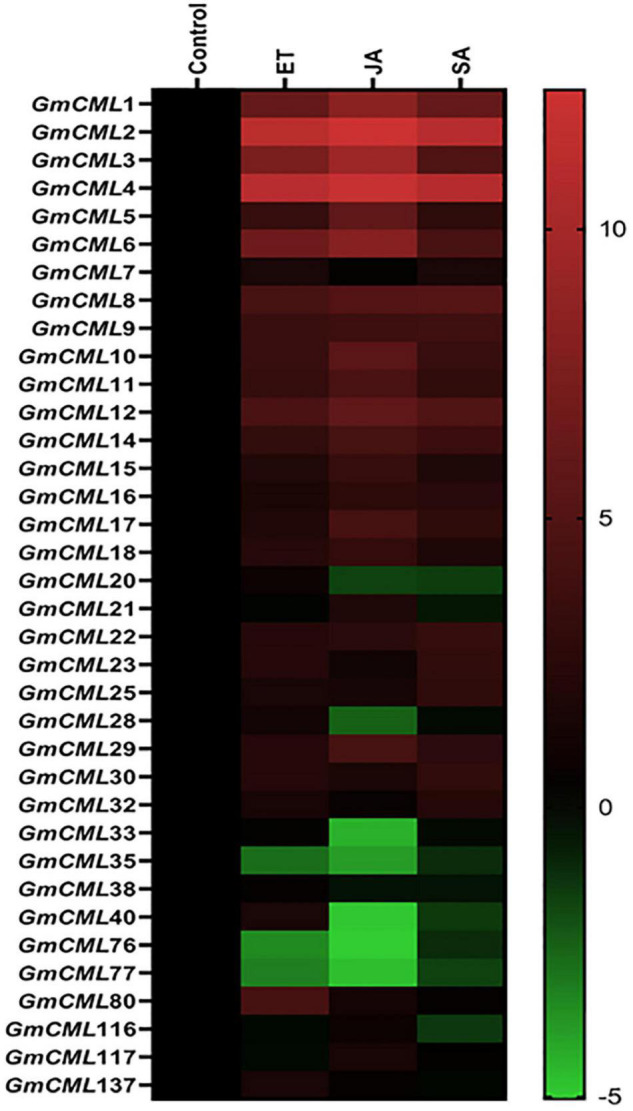
Heat map showing expression profile of *GmCML*s on application of JA, SA, and ET. The expression profile of *GmCML*s on application of JA, SA, and ET is represented as a heat map, generated with the help of Graphpad Prism software using log^2^ values of expression data obtained from qPCR. The qPCR data were analyzed utilizing the 2^–ΔΔCT^ method and statistical analysis was performed using ANOVA and Tukey’s test (*P* < 0.05).

To recognize the role of defense-related signaling compounds in the regulation of *S. litura* inducible-*GmCMLs*, expression analysis was performed with plants treated with JA, SA, and ethylene (ET) by qPCR. *GmCMLs* 1, 2, 3, 4, 5, 6, 8, 9, 10, 11, and 12 showed induced expression in all four treatments. The JA application also induced the expression of *GmCMLs* 14, 17, and 29. The ethylene upregulated the expression of 13 of the *GmCMLs*, and eight of them were commonly upregulated by JA and ethylene. In addition, eleven of the *GmCML*s were upregulated by SA (Figure 3; Supplementary Figure 2).

### Subcellular Localization, Molecular Weight, Isoelectric Point, and EF-Hand Analysis of *Glycine max* CMLs

The subcellular localization study using *in silico* tools predicted GmCMLs to be located mainly in the nucleus, cytosol, or chloroplast. Only a few of them were predicted to be located on the plasma membrane. The GmCMLs ranged from 229 (GmCML22) to 102 (GmCML38) amino acids, and their molecular weight ranged between 11.26 and 25.85 KDa. GmCML38, 32, and 11 were smallest by molecular weights (11.26 KDa), whereas GmCMLs 22 and 25 were of the largest molecular weight (25.85 KDa). The isoelectric point values of the GmCMLs ranged from pI 3.94 to 6.84, where GmCML2 had a minimum isoelectric point (pI 3.94) and GmCML80 had a maximum isoelectric point (pI 6.84) (Table 1).

**TABLE 1:** Gene locus, chromosome number, number of amino acids, number of EF-hands, molecular weight, pI, and subcellular localization of *S. litura*-inducible GmCMLs.

Gene name	Locus	Chromosome	Amino acid	Number of EF-hands	MW (kDa)	pI	PSORT subcellular localization
GmCML1	Glyma.10G178400	Chr10	150	4	17.01	4.04	chlo: 5, cyto: 5, extr: 2, cysk_nucl: 1.33333, cysk_plas: 1.33333
GmCML2	Glyma.02G002100	Chr02	150	4	17	3.94	cyto: 6.5, cyto_nucl: 4.5, chlo: 4, extr: 2, nucl: 1.5
GmCML3	Glyma.20G211700	Chr20	150	4	17	4.03	chlo: 5, cyto: 5, extr: 2, cysk_nucl: 1.33333, cysk_plas: 1.33333
GmCML4	Glyma.10G002200	Chr10	150	4	16.89	4.01	cyto: 7, cyto_nucl: 6.5, chlo: 4, extr: 1
GmCML5	Glyma.13G074800	Chr13	140	4	15.81	4.15	cyto: 6, nucl: 3, extr: 3, chlo: 1, golg: 1
GmCML6	Glyma.20G048900	Chr20	165	4	18.77	4.6	chlo: 12, mito: 2
GmCML7	Glyma.19G244300	Chr19	149	4	17.09	4.09	cyto: 10, nucl: 2, golg: 2
GmCML8	Glyma.19G160100	Chr19	148	4	16.7	4.17	cyto: 11, extr: 2, nucl: 1
GmCML9	Glyma.03G157800	Chr03	148	4	16.86	4.16	cyto: 14
GmCML10	Glyma.03G246800	Chr03	149	4	17.07	4.12	cyto: 10, nucl: 2, extr: 1, golg: 1
GmCML11	Glyma.19G098900	Chr19	114	2	13.25	4.15	cyto: 7, chlo: 3, nucl: 2, extr: 2
GmCML12	Glyma.10G161900	Chr10	149	4	17.21	4.32	cyto: 6, cyto_nucl: 5.83333, cyto_E.R.: 4, chlo: 3, nucl: 2.5, extr: 1, golg: 1
GmCML14	Glyma.20G224300	Chr20	149	4	17.2	4.35	cyto_nucl: 5.83333, cyto: 5, nucl: 3.5, cyto_E.R.: 3.5, chlo: 3, extr: 1, golg: 1
GmCML15	Glyma.02G143800	Chr02	149	4	17.19	4.28	cyto: 12, extr: 1, golg: 1
GmCML16	Glyma.10G030500	Chr10	149	4	17.12	4.25	cyto: 13, golg: 1
GmCML17	Glyma.11G030100	Chr11	147	4	16.55	4.73	cyto: 5, nucl_plas: 4, nucl: 3.5, plas: 3.5, chlo: 1, extr: 1
GmCML18	Glyma.01G211700	Chr01	147	4	16.54	4.75	cyto: 8, plas: 3, chlo: 1, nucl: 1, extr: 1
GmCML20	Glyma.17G134900	Chr17	147	4	16.55	5.66	cyto: 5, nucl: 2, plas: 2, extr: 2, pero: 1, cysk: 1, golg: 1
GmCML21	Glyma.05G238400	Chr05	188	4	20.43	4.28	nucl: 9, chlo: 3, mito: 1, extr: 1
GmCML22	Glyma.17G112000	Chr17	229	4	25.83	4.63	chlo: 9, cyto: 2, nucl: 1, extr: 1, E.R.: 1
GmCML23	Glyma.01G094000	Chr01	152	4	16.38	4.05	cyto: 6, chlo: 4, extr: 2, golg: 1, cysk_nucl: 1
GmCML25	Glyma.13G159600	Chr13	229	4	25.58	4.75	chlo: 7, extr: 3, vacu: 2, cyto: 1, cysk_nucl: 1
GmCML28	Glyma.11G217200	Chr11	137	4	15.49	5.07	mito: 8, nucl: 3, chlo: 1, cyto: 1, extr: 1
GmCML29	Glyma.14G215800	Chr14	141	4	15.89	4.62	cyto: 6, mito: 6, nucl: 1, extr: 1
GmCML30	Glyma.03G127000	Chr03	152	4	17	4.32	nucl: 4, chlo: 3, cyto: 3, mito: 2, extr: 2
GmCML32	Glyma.11G127500	Chr11	106	3	12.03	4.12	chlo: 3, extr: 3, cysk_nucl: 3, nucl: 2.5, cysk: 2.5, cyto: 2, golg: 1
GmCML33	Glyma.02G245700	Chr02	141	4	15.89	4.62	mito: 9, chlo: 3, nucl: 1, extr: 1
GmCML35	Glyma.19G129800	Chr19	152	4	16.95	4.25	nucl: 5, extr: 4, chlo: 2, ito: 2, cyto: 1
GmCML38	Glyma.09G067600	Chr09	102	2	11.26	4.08	cyto: 7, chlo: 3, extr: 2, nucl: 1, cysk: 1
GmCML40	Glyma.13G344200	Chr13	184	4	21.26	4.98	chlo: 9, mito: 3, cyto: 2
GmCML76	Glyma.09G270900	Chr09	183	3	20.52	4.48	mito: 8, chlo: 4, nucl: 2
GmCML77	Glyma.16G059300	Chr16	140	4	15.96	4.49	nucl: 6, chlo: 5, extr: 2,plas: 1
GmCML80	Glyma.07G212000	Chr07	185	4	21.2	6.84	chlo: 5, mito: 5, nucl: 4
GmCML116	Glyma.09G236800	Chr09	207	2	24.06	4.44	chlo: 5, nucl: 4, extr: 2, cyto: 1, cysk: 1, golg: 1
GmCML117	Glyma.18G260700	Chr18	207	3	24.08	4.51	chlo: 5, nucl: 5, extr: 2, cyto: 1, golg: 1
GmCML137	Glyma.04G078400	Chr04	178	4	19.71	4.61	nucl: 5, cyto: 5, chlo: 3, extr: 1

### *GmCMLs* Are Largely Intronless/Carry 1–3 Introns and Share EF-Hand Signature Sequence and Other Common Motifs

**FIGURE 4:**
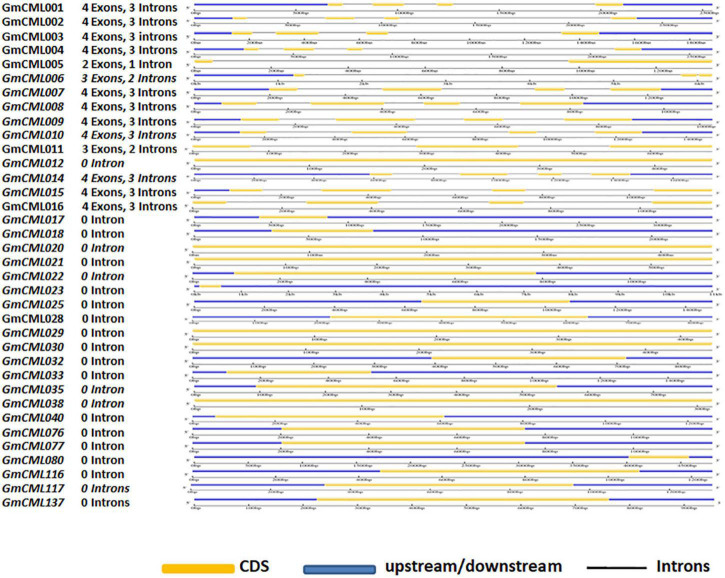
The gene structure analysis of *GmCML*s. The gene structure analysis showing the distribution of exon-intron in *GmCMLs*. The exons, upstream/downstream, and introns are represented by yellow, blue, and black colors, respectively. The exon-intron distribution analysis was examined using Gene Structure Display server GSDS2.0.

**FIGURE 5:**
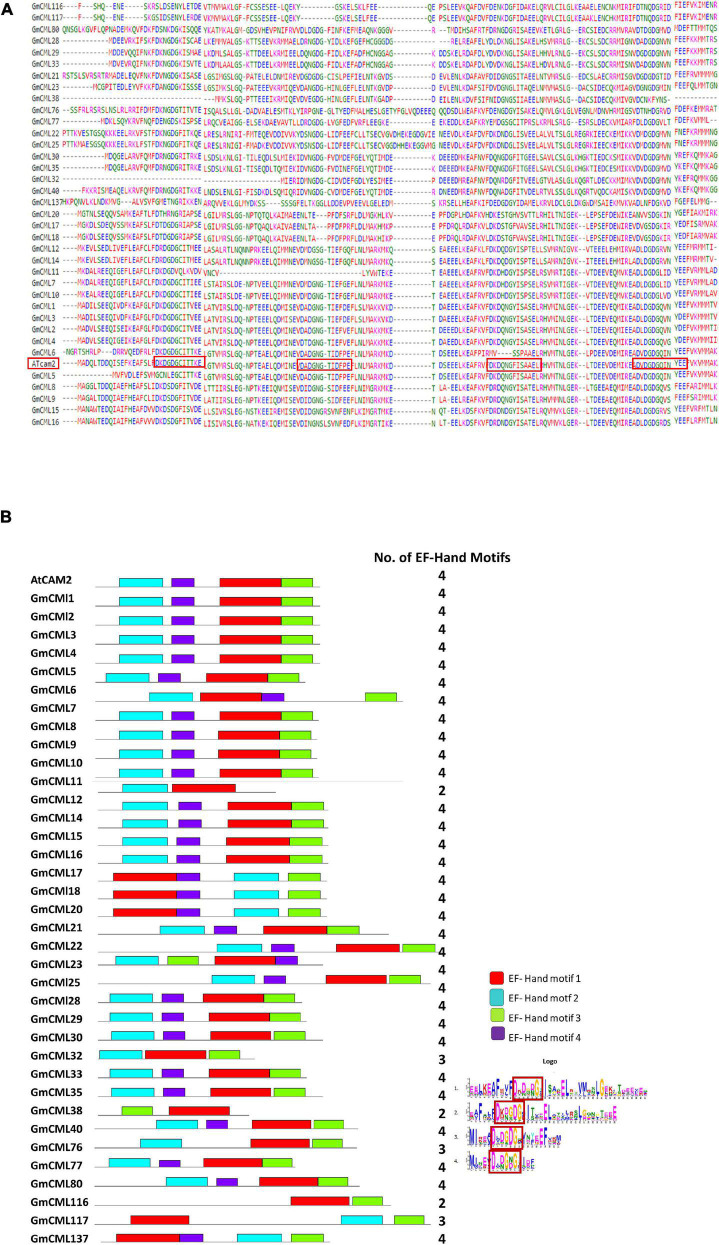
Identification of EF-hands in GmCMLs by *in silico* method. The presence of EF-hands (number varied from 2 to 4) in GmCMLs are presented **(A)**. The boxes represent the EF-hands present in each GmCMLs. The logoplot shows the presence of respective number of EF-hands in GmCMLs (generated by MEME suite) **(B)**. The colored boxes indicate the number of EF-hand motifs at the corresponding position within GmCMLs. Different colors indicate different EF-hand motifs.

The intron and exon display analysis of *GmCMLs* revealed that a larger number of these CMLs were intronless. Among the studied 36 *GmCMLs*, 22 *GmCMLs* coding genes were intronless, whereas the rest of the *GmCML*s had one to three introns. *GmCML*s 1, 2, 3, 4, 7, 8, 9, 10, 14, 15, and 16 have four exons and three introns (Figure 4). Additionally, prediction of the conserved domain revealed that a couple of motifs were conserved among the members of GmCML family proteins. Many of the GmCMLs (30 GmCMLs) had four EF-hands but GmCML 32, 76, and 117 had three EF-hands and GmCML11, 38, and 137 had only two EF-hands (Figure 5). All GmCML proteins contained the EELKEAFKVF**DKDGBGYISASE**LRHVMRSLGEKLTDEEVEQ, EAFSLF**DKBGDGCITVEE**LATIJRSLGQNPTEEE, and MIKEA **DLDGDGQVBYEE**FVKM motif confirming the presence of EF-hand signature sequence.

### Members of *Glycine max CMLs* Family Differ in Their Tissue-Specific Expression Patterns

**FIGURE 6:**
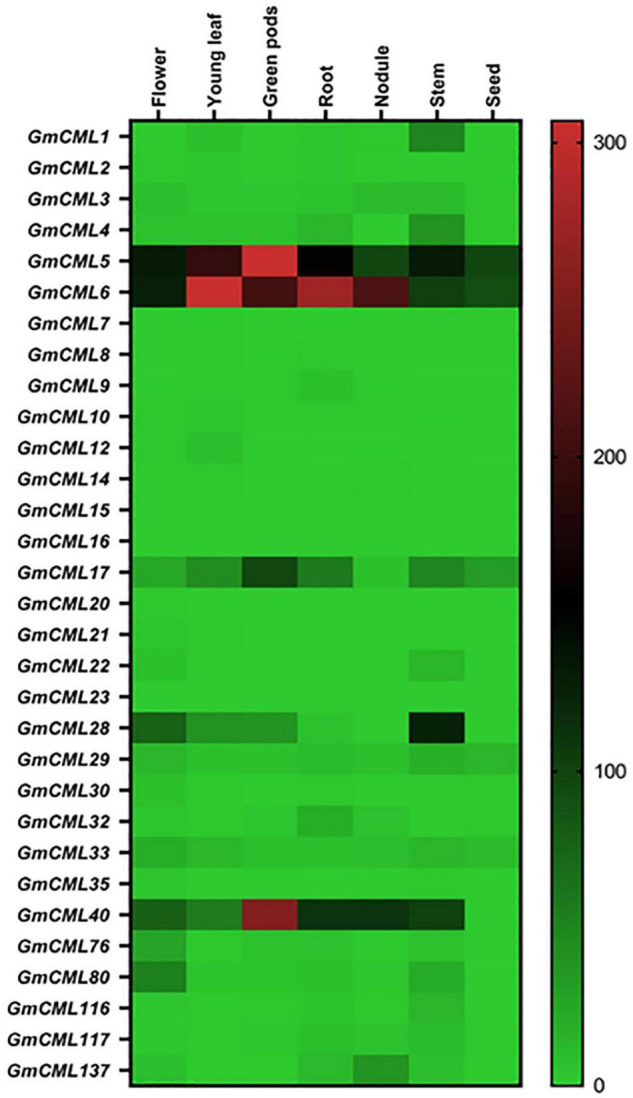
The expression profile of *GmCML*s in different tissues of soybean. Heat map showing expression profiles of *GmCMLs* in different tissue types of *G. max*. The RPKM values were obtained from SoyBase Expression Explorer. The heat map has been generated using Graphpad Prism software. Expression data of *GmCML011, GmCML018, GmCML025, GmCML038*, and *GmCML077* were not available.

To identify the genes involved in distinct regulatory functions controlled by specific tissue, tissue-specific gene expression data were procured. The heat map was generated using RNA-seq data to examine the tissue-specific expression profile at the basal level (Figure 6; Supplementary Table 3). This analysis revealed that the relative expression level of *GmCMLs* 5, 6, and 40 was higher in all tissue types. *GmCMLs* 6, 5, 40, 28, 17, 80, 1, 4, and 29 showed their higher expression in stem. *GmCML*s 5, 6, and 17 showed expression in seed and *GmCMLs* 6, 5, 40, 17, 80, and 137 expressed more in the nodule. *GmCML*17 had moderate expression in all the tissues, including flower, young leaf, green pods, root, nodule, stem, and seed. *GmCML*s 6, 5, 40, 28, 17, 80, 1, 4, 29, 33, 76, and 32 exhibited maximum expression in root. *GmCMLs* 1, 6, 5, 40, 28, 17, and 80 exhibited higher expression in leaf. The mRNA of *GmCML*s 6, 5, 40, 28, 17, and 80 were abundantly present in flower and pod. However, the rest of the genes showed minimal expression/no expression in different tissues.

### Members of *Glycine max CML* Family Are Putative Targets of Known microRNAs

The miRNA target analysis (using psRNATarget) revealed 33 *GmCML* transcripts as putative targets of 125 miRNAs (Table 2).

**TABLE 2:** Identification of miRNA target sites in *GmCML*-transcripts of *Glycine max.*

miRNA_Acc.	Target_Acc.	Number of miRNA targeting target gene
gma-miR171k-5p	Glyma.01G211700	3
gma-miR4352b	Glyma.02G002100	3
gma-miR1517	Glyma.02G143800	2
gma-miR159a-5p	Glyma.02G245700	3
gma-miR5376	Glyma.03G127000	1
gma-miR5667-5p	Glyma.03G157800	1
gma-miR167a	Glyma.03G246800	10
gma-miR172a	Glyma.04G078400	12
gma-miR159a-5p	Glyma.05G238400	2
gma-miR172a	Glyma.06G079900	14
gma-miR4370	Glyma.07G169100	5
gma-miR396a-5p	Glyma.07G212000	11
gma-miR4400	Glyma.09G236800	4
gma-miR4397-3p	Glyma.09G270900	3
gma-miR4352b	Glyma.10G002200	3
gma-miR5667-5p	Glyma.10G030500	2
gma-miR408d	Glyma.10G178400	2
gma-miR2119	Glyma.11G030100	4
gma-miR1514b-5p	Glyma.12G052100	6
gma-miR1515a	Glyma.13G074800	4
gma-miR4347	Glyma.13G159600	2
gma-miR4368a	Glyma.13G344200	1
gma-miR159a-5p	Glyma.14G215800	3
gma-miR4347	Glyma.17G112000	3
gma-miR171k-5p	Glyma.17G134900	2
gma-miR159a-5p	Glyma.18G039500	2
gma-miR396f	Glyma.18G260700	3
gma-miR5040	Glyma.19G098900	1
gma-miR4384	Glyma.19G129800	2
gma-miR1517	Glyma.19G160100	3
gma-miR5040	Glyma.19G244300	2
gma-miR1515a	Glyma.20G048900	4
gma-miR6299	Glyma.20G211700	2

### Members of *Glycine max CMLs* Family Have an Overlapping Protein Network

**FIGURE 7:**
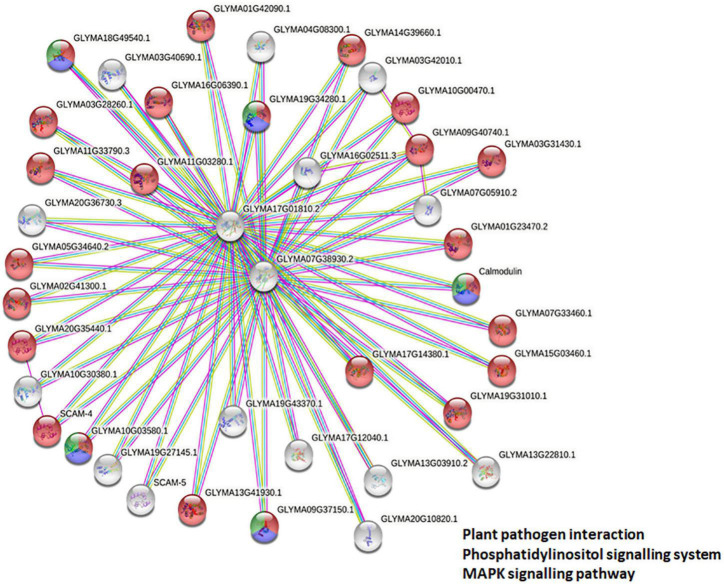
Protein-protein interaction network analysis. Protein-protein interaction network and enrichment analysis showing the complex interplay among GmCMLs as predicted from the STRING database. The proteins colored in red are showing their potential involvement in plant-pathogen interaction, the proteins in blue color are predicted to be involved in phosphatidylinositol signaling, and the proteins in green color are predicted to be involved in MAPK signaling pathways.

To deduce the complex interplay of GmCMLs with other proteins, network and enrichment analysis of 36 selected GmCMLs was performed using the STRING database. The studies revealed that each GmCML corresponded to a particular string Id. Based on the STRING analysis, a protein protein interaction (PPI) network complex was constructed. The expected number of edges of the interaction network was 6, the average node degree of the network was 4, the average local clustering coefficient was 0.986, and the protein enrichment *p*-value was < 1.0e-16. The predicted results showed 40 nodes and 80 edges. Out of 36, 25 of the GmCMLs had shown their involvement as calcium-binding protein having EF-hand, where GmCML1, GmCML2, and GmCML15 had shown very high similarity with SCAM-4, SCAM-5, and Calmodulin, respectively, which were reported to be involved in plant-defense against pathogen attack (Park et al., 2004). Interestingly, the studies have shown the involvement of GmCML5 and GmCML6 in nodulation of soybean roots. Most of the GmCMLs showed to interact *via* GLYMA07G38930.2 (spindle-like microcephalyassociated protein isoform) and GLYMA17G01810.2 (spindle-like microcephalyassociated protein isoform). Overall, it was predicted that 24 GmCMLs have their role in plant-pathogen defense signaling and 5 of them showed their role in the phosphatidylinositol signaling and MAPK pathway (Figure 7).

### *In silico* Promoter Analysis Identified Multiple Hormones and Stress-Responsive *Cis*-Elements in Promoter Sequences of *Glycine max CMLs*

**FIGURE 8:**
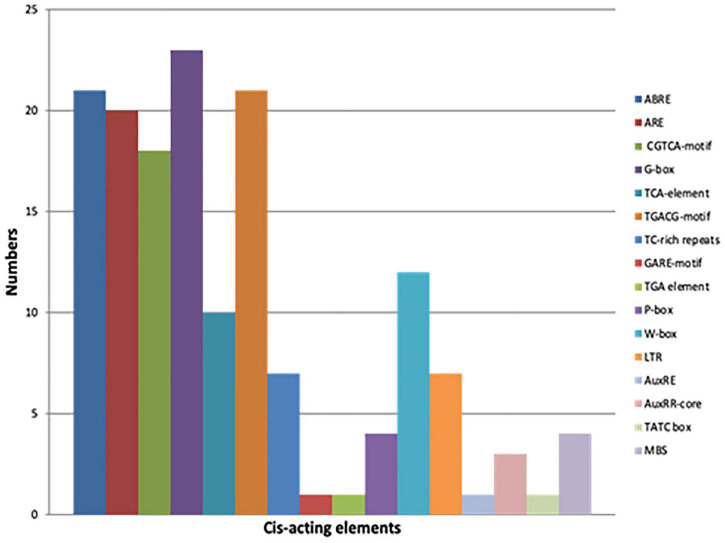
The graphical representation of different *cis*-acting elements present in the promoter sequences of *GmCMLs.* Different colors depict different *cis*-acting elements [ABRE – Abscisic acid responsiveness, ARE –Anaerobic induction (wounding), CGTCA motif – Jasmonate responsiveness (MeJA), TCA-element – Salicylic acid responsiveness, TGACG-motif - Jasmonate responsiveness (MeJA), TC-rich repeats –defense and stress response, GARE-motif – Gibberellin responsiveness, TGA element – Auxin response, P Box - Gibberellin responsiveness, W-Box - Stress inducible, LTR – Low temperature response, AuxRE- Auxin responsive element, AuxRR-core - Auxin responsive element, TATC box - Gibberellin responsiveness and MBS – Drought inducible].

A broad range analysis to decipher which *cis*-elements were present in the promoter regions of true *GmCMLs*, 1 kb 5′ UTR regions of each of the *GmCMLs* were subjected to Plant care analysis that predicted the presence of 16 different *cis*-regulatory elements involved in biotic or abiotic stresses. Most of the *GmCMLs* promoters were predicted to harbor more than one phytohormone-related *cis*-regulatory elements, such as ABA (ABRE), JA (G-box, Myc binding JA response element), SA (TCA-element), Ethylene (ERE), GA (P-Box and GARE-motif – gibberellin responsiveness), and biotic and abiotic stress-related *cis*-elements like ARE (Anaerobic induction), wounding, Myb-binding site involved in drought-inducibility (MBS), W-box-stress inducible and LTR (a low temperature)-responsive element, TC-rich repeats –defense and stress response (Figure 8).

### Molecular Docking Indicates That Members of *Glycine max CML* Family Proteins Interact With Ca^2+^

**FIGURE 9:**
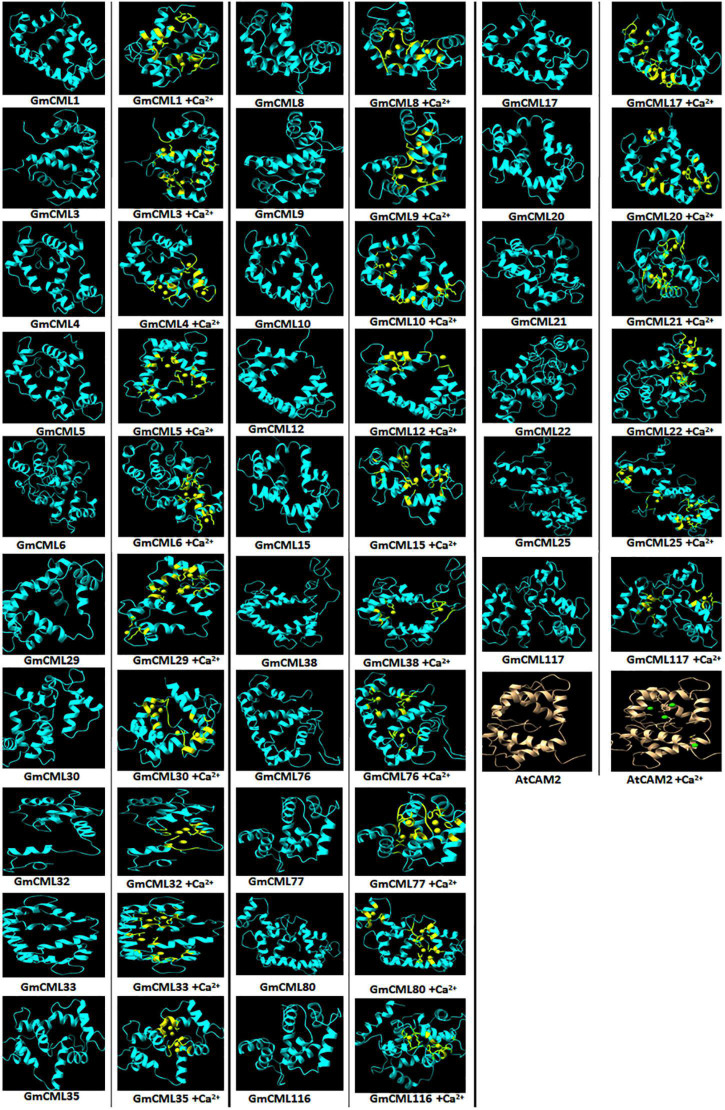
Interaction of GmCMLs with Ca^2+^ using molecular docking and their comparison with interaction of Calmodulin 2 (*A. thaliana*) with Ca^2+^. The figure shows the modeled tertiary structures of GmCMLs and their interaction with Ca^2+^, wherein the yellow balls indicate Ca^2+^ and the yellow-colored segments in the model represent EF-hands. The number of Ca^2+^ showing interaction with GmCMLs varied according to the number of EF-hands. The global docking energy score of interaction between 26 GmCMLs with Ca^2+^ is shown to have near to global docking energy score of interaction between Calmodulin 2 of *A. thaliana* with Ca^2+^. The tertiary structure of *A. thaliana* is shown in gray color and the green balls indicate Ca^2+^.

The study of the interaction of Ca^2+^ with GmCMLs could be crucial for understanding their mechanism of action. The tertiary structure of GmCMLs was modeled using Phyre^2^, and the structures were validated by keeping a threshold value of >90% region of protein being present in the allowed region of the Ramachandran plot. The confidence level of modeled structures was more than 99%. The classic EF-hands consisting of a typical helix-loop-helix domain were detected and highlighted (Figure 9).

Previous studies had indicated that EF-hand motifs bind with Ca^2+^ for their activation. Thus, the molecular docking of 36 GmCMLs and Calmodulin 2 protein of *Arabidopsis thaliana* (AtCAM2) with Ca^2+^ were performed, and the interaction results of GmCMLs with Ca^2+^ were compared with the global energy score of AtCAM 2 with Ca^2+^. The global energy score of AtCAM2 with Ca^2+^ was −2.94. The global energy score for 26 of the GmCMLs with Ca^2+^ ranged from −2.5 to −4.5. GmCMLs 17, 15, 29, 38, 116, 117, 33, 25, 30, 8, 35, 21, 76, 32, 77, 6, 22, 5, and 80 were showing the global energy scores from −4.41 to −3.05 whereas GmCMLs 20, 9, 12, 1, 10, 3, and 4 were showing global energy scores in between −2.8 and −2.5 (Figure 9).

## Discussion

Plants have to encounter numerous challenges ranging from biotic to abiotic factors during their lifespan. Therefore, they have evolved sophisticated defense mechanisms regulated by recognizing the specific type of stress and stimulation of certain early signaling events to defend themselves. One such crucial early event is the generation of Ca^2+^ ion pulses due to the elevation of influx and efflux, which acts as a cellular signal in response to external stimulus (Vadassery et al., 2012a,b). Calcium sensor proteins, such as CMLs, are essential components of Ca^2+^ signaling (DeFalco et al., 2010). We have only a little information about the involvement of CMLs in plant defense against herbivory in crop plants. Although fewer studies on the EF-hand possessing protein families, CaM, CDPK, CMLs, and CBLs (Weinl and Kudla, 2009; DeFalco et al., 2010; Poovaiah et al., 2013) have been taken into consideration in a couple of plants, such as *Arabidopsis thaliana, Oryza sativa*, Grapevine, *Nicotiana benthamiana, and Solanum lycopersicum* but significantly less information are available about CMLs in legumes (McCormack and Braam, 2003; Asano et al., 2005; Boonburapong and Buaboocha, 2007; Chen et al., 2013; Kong et al., 2013; Kleist et al., 2014).

Therefore, to characterize the CMLs of soybean and figure out their role in plant defense, the GmCMLs were subjected to phylogenetic analysis with CMLs of *Medicago truncatula*, *Arabidopsis thaliana*, and *Oryza sativa*, which revealed that many of the GmCMLs were grouped with CMLs of *Arabidopsis* and *Medicago*. They shared ancestry, which confirmed their evolutionary relationship. However, when GmCMLs were subjected to the BLAST search engine of TAIR against the known EF-hand protein of *A. thaliana*, only 41 GmCMLs had more than 50% sequence similarity. Expression analysis of true *GmCMLs* during *S. litura*-infestation was investigated, and the results demonstrated that many of these *GmCMLs* (36) were responsive to herbivory. Out of 41 genes, 34 of the *GmCMLs* were upregulated, while 2 of the *GmCMLs* were downregulated. Previous studies have reported that *CMLs* control signaling events upregulated during mechanical wounding and insect attack. It was observed that in *A. thaliana, CML9, 11, 12, 16, 17, 23*, and *42* genes were upregulated upon treatments with the oral secretion of larvae of the herbivorous insect *S. littoralis* (Vadassery et al., 2012a). In comparison to herbivory, 24 *GmCMLs* were upregulated 5 *GmCMLs* were downregulated by mechanical damage, and 8 of the *GmCMLs* showed induced expression exclusively during *S. litura*-infestation. These data suggested that wounding and herbivory had a different transcript pattern on account of the presence of insect-derived elicitors in the oral secretion during herbivory. The distinction in the transcript patterns during mechanical damage and insect attack has been detected in previous studies (Reymond et al., 2000, 2004; Singh et al., 2008). Wounding stimulated plant defense similar to herbivory; however, mechanical damage-induced responses were not the same as those activated by herbivory.

Signaling compounds, such as JA, ET, and SA, function as essential plant defense compounds against herbivory. Most of the herbivores trigger the jasmonate/ethylene pathway and the salicylate pathway (Hammerschmidt and Smith-Becker, 1999; Reymond et al., 2004). To recognize the role of defense regulators, change in the expression of *GmCMLs* was checked upon treatments with JA, SA, and ET. JA changed the mRNA levels of 14 of the *S. litura*-inducible *GmCMLs*, demonstrating the involvement of JA in the regulation of expression of *GmCMLs*. JA-responsive *cis*-regulatory elements, TGACG and CGTCA, were observed in the promoter regions of *GmCML*s 1, 3, 9, 14, 7 and 1, 3, 9, 14, 17, respectively, which further confirmed their regulation by JA. Ethylene upregulated the expression of 13 of the *GmCMLs*, and eight of them were commonly upregulated by JA. ET and JA were reported to induce defense genes synergistically in *Arabidopsis* (Kessler and Baldwin, 2002; Singh et al., 2008). In addition, eleven of the *GmCML*s were upregulated by SA, indicating the role of SA in regulation of expression of *GmCMLs* either directly or through a crosstalk. The presence of SA-responsive *cis*-regulatory element in the promoter of *GmCML* 8 further confirmed their regulation by SA.

The exon-intron distribution study predicted the presence of zero to three introns in *GmCMLs*, indicating critical evolutionary changes in the *G. max* genome. There were only 11 *GmCMLs* that carried 1–3 introns, and 22 *GmCMLs* were intronless. The presence of few/no introns indicated their ability to get transcribed quickly to facilitate early defense response in the host plant during stress (Keshan et al., 2021).

The subcellular localization studies predicted the presence of CMLs in the nucleus, cytosol, and chloroplast mainly. Previous studies on CMLs had reported their localization in the nucleus and cytosol in *Arabidopsis* (Inzé et al., 2012; Vadassery et al., 2012a). The lower values of isoelectric points revealed that *GmCMLs* could be acidic, making them highly hydrophilic. The motif analysis exhibited 1 to 4 EF-hand potential motifs in most of the GmCMLs, indicating their potential identity as calcium-binding proteins (Boonburapong and Buaboocha, 2007; Kong et al., 2013; Kleist et al., 2014; Munir et al., 2016). Further, identifying potential miRNA target sites in *GmCMLs* divulged the dynamic roles of miRNA in post-transcriptional regulation of these genes in response to normal or stressed conditions (Bhatia et al., 2019).

The promoter of every gene possesses regulatory elements that act as binding sites for transcription factors. These promoter elements govern the differential expression of a gene in a tissue-specific manner at different growth stages and during environmental stresses. Several studies have divulged a direct connection between gene expression and promoter elements present in their 5′ upstream region (Lata et al., 2014). Therefore, *cis*-elements present in the promoter regions of all true *GmCMLs* were investigated using *in silico* tool, which predicted the presence of 16 different *cis*-regulatory elements involved in biotic or abiotic stresses, indicating their role in plant responses during stress and their involvement in signal transduction *via* ABA, SA, and JA.

The analysis of measuring mRNA abundance of genes belonging to a family in a tissue-specific manner will allow in identifying the genes involved in the development or regulatory pathways associated with a particular tissue type. The expression data of the *GmCML* gene family in different organs exhibited divergence in their function. The transcript profiling of *GmCMLs* during several developmental stages in different organs revealed that most *GmCMLs* do not express vegetatively. They are inducible to exhibit a particular response, whereas some *GmCML*s, such as *GmCML*5, *GmCML*6, and *GmCML*40, expressed highly in flower, root, pod, pod shell, young leaf, and seed, depicting their role in growth and development. Nine of the *GmCMLs* exhibited higher expression in the stem, illustrating their potential role in shoot development. Three of the *GmCMLs* showed their expression in seed, and six showed their expression in the nodule, indicating their prospective role in seed and nodule formation. Twelve *GmCMLs* showed higher expression in root, signifying their possible function in root development and plant-microbe interaction in soil. Seven of the *GmCMLs* exhibited higher expression in the leaf, specifying their role in leaf development and plant defense against pests and pathogens. Six of the *GmCMLs* were expressed abundantly in flower and pod, depicting their role in reproduction.

To deduce the complex interplay of GmCMLs with other proteins, network and enrichment analysis of *S. litura*-inducible 36 GmCMLs were performed with the STRING database. The PPI network complex indicated that out of 36 GmCMLs, 24 GmCMLs were predicted to play a role in plant-pathogen defense signaling; 5 of the GmCMLs (GmCML16, GmCML8, GmCML15, GmCML116, and GmCML117) could be involved in the phosphatidylinositol signaling system and MAPK signaling system. The plant defense signaling, phosphatidylinositol signaling system, MAPK pathway, and the components of these pathways, such as SCAM-4, SCAM-5, CaMEKK, CaZIK, CaRAF, PLA, PLC, and PLD had been reported to be involved in plant defense against biotic stresses (Park et al., 2004; Hung et al., 2014; Keshan et al., 2021). The above results are prediction-based and should be perceived with caution. Further empirical analyses and experimentation would be required to assess whether these interactions are physiologically relevant.

The interaction of CMLs with Ca^2+^ is crucial for their biological activity. To examine the interaction between Ca^2+^ and GmCMLs, the tertiary structure of *S. litura*-inducible GmCMLs was predicted, which demonstrated a difference in the modeled structures of GmCMLs, mainly due to the change in their amino acid sequences. This diversity in their structures could allow them to perform a specific function. However, all the modeled structures of GmCMLs had shown the EF-hand helix-loop-helix motif as described in Calmodulin of *A. thaliana*, which could facilitate their interaction with Ca^2+^. The molecular docking was performed to investigate the interaction of GmCMLs with Ca^2+^ and their global energy score of interaction was compared with the global energy score of interaction between AtCAM2 with Ca^2+^. The results revealed that the global energy score for interaction between GmCMLs with Ca^2+^ ranged from −2.5 to −4.5 (for 26 of the GmCMLs), which is comparable to AtCAM2 with Ca^2+^ interaction. Thus, *in silico* interaction study predicted that calcium ions interacted with GmCMLs, particularly with EF-hand motifs, indicating that GmCMLs could be Ca^2+^-binding proteins and could be involved in Ca^2+^ signaling. Further experimentation would be required to confirm whether CMLs are undergoing any conformational change to induce downstream signaling.

## Conclusion

**FIGURE 10:**
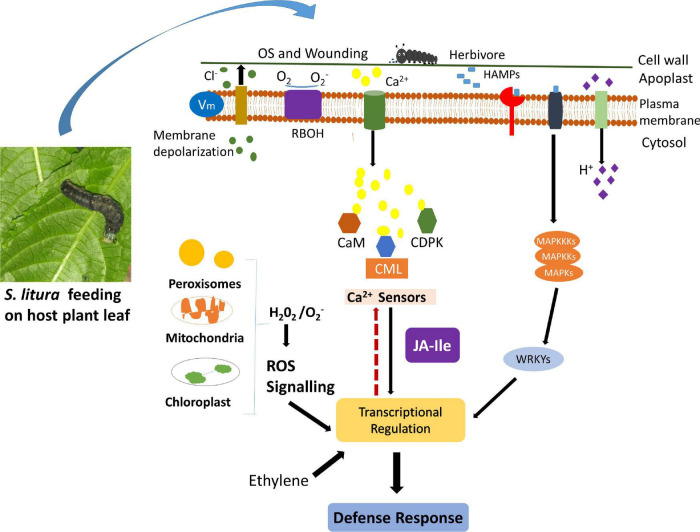
Schematic representation of herbivory-induced early signaling events (calcium signaling, ROS generation, activation of MAPK Pathway) and involvement of CMLs in plant defense response against insect attack. Upon herbivory, plants perceive stimuli and instigate a couple of early events, such as activation of calcium signaling, induction of mitogen-activated protein kinase pathway, and generation of reactive oxygen species, which transduce the signal and activate the biosynthesis of other signaling and defensive compounds. In soybean, the expression of few GmCMLs, one of the crucial Ca^2+^ sensors, is upregulated during *S. litura*-infestation. GmCMLs have Ca-EF hands, which allow them to sense calcium signature and bind with Ca^2+^, which can further regulate downstream signaling and activate plant defense response.

Plants encounter various environmental stresses, including insect attack during their life span. To defend themselves, plants have developed sophisticated signaling pathways that activate the biosynthesis of defensive proteins and metabolites. Among a couple of early events that instigate defense signaling, a transient change in cytosolic Ca^2+^ concentration is observed during herbivory. This transient change in Ca^2+^ concentration is identified by Ca^2+^ sensors, which transduce the information to downstream target proteins leading to appropriate defense response. The role of Ca^2+^ sensors during herbivory has been studied in model plants, but not in leguminous plants, such as *G. max*. This study identified 41 true *GmCMLs*, an important Ca^2+^-sensor, in soybean. The phylogenetic analysis revealed their similarity with already known *CMLs* from model plants. The presence of potential EF-hands further supported their identification as Ca^2+^-sensors. The gene structure determination of *GmCMLs* indicated the presence of no/few introns revealing their capability to change their expression quickly during recognition of a stimulus. The differential expression of *GmCMLs* in various tissues signified their involvement in growth and development. Alteration in the transcript patterns of the novel *GmCMLs* during *S. litura*-infestation suggested their significant involvement in plant defense signaling during herbivory (Figure 10). Comparative expression profiling during mechanical damage showed that wounding-regulated responses are not the same as observed during herbivory. Signaling compounds, such as JA, ET, and SA, showed their involvement in regulating the expression of *S. litura*-inducible *GmCMLs.* Network and enrichment analysis of GmCMLs with other proteins indicated their role in plant defense, phosphatidylinositol signaling system, and MAPK signaling system. The study on the interaction between calcium and GmCMLs showed their identity as Ca^2+^-binding proteins and their participation in signal transduction during Ca^2+^ signaling (Figure 10). The results of this study can be extended to confirm the role of calcium signaling in triggering the plant defense mechanism against *S. litura*-infestation. *S. litura*-inducible *GmCMLs* are prospective candidates for being critical components of calcium signaling during pest attacks. Thus, the structural and functional characterization of *S. litura*-inducible *GmCMLs* utilizing contemporary techniques like RNAi, overexpression, and/or gene editing is highly recommended to delineate the calcium signaling pathway and its role in plant defense during soybean-*S. litura* interaction.

## Data Availability Statement

The original contributions presented in the study are included in the article/Supplementary Material, further inquiries can be directed to the corresponding authors.

## Author Contributions

AS conceptualized and supervised the study. MY, JP, AS, IKS, AC, AR, MH, and JKK contributed to the investigation. MY, JP, and AS wrote – original draft preparation. AS, IKS, AC, AR, MH, and JKK contributed to writing, reviewing, editing, and visualization. AS, IKS, and MY contributed to formal analysis. AS, AC, and AR contributed to funding acquisition. IKS and AS contributed to resources. All authors have read and agreed to the published version of the manuscript.

## Conflict of Interest

The authors declare that the research was conducted in the absence of any commercial or financial relationships that could be construed as a potential conflict of interest.

## Publisher’s Note

All claims expressed in this article are solely those of the authors and do not necessarily represent those of their affiliated organizations, or those of the publisher, the editors and the reviewers. Any product that may be evaluated in this article, or claim that may be made by its manufacturer, is not guaranteed or endorsed by the publisher.

## Footnotes

1
https://www.soybase.org/

2
https://phytozome.jgi.doe.gov/pz/portal.html

3
https://blast.ncbi.nlm.nih.gov/Blast.cgi

4
https://www.ncbi.nlm.nih.gov

5
https://www.arabidopsis.org/

6
https://shigen.nig.ac.jp/rice/oryzabase/

7
https://www.ebi.ac.uk/Tools/msa/clustalo/

8
https://itol.embl.de/itol.cgi

9
https://www.arabidopsis.org/Blast/

10
http://bioinfo.ut.ee/primer3-0.4.0/

11
https://www.graphpad.com/scientific-software/prism

12
https://www.genscript.com/wolf-psort.html

13
https://web.expasy.org/compute_pi/

14
https://www.ebi.ac.uk/interpro/

15
http://meme.sdsc.edu/meme/meme.html

16
http://gsds.gao-lab.org/

17
https://www.soybase.org/expression/

18
http://plantgrn.noble.org/psRNATarget/

19
https://string-db.org/cgi/input?sessionId=bcguyAaLcQA5&input_page_show_search=on

20
http://bioinformatics.psb.ugent.be/webtools/plantcare

21
https://pubchem.ncbi.nlm.nih.gov/

22
http://www.cheminfo.org/Chemistry/Cheminformatics/FormatConverter/index.html

23
https://bioinfo3d.cs.tau.ac.il/FireDock/
