# Supplementary material for: A Comprehensive Analysis of Calmodulin-Like Proteins of Glycine max Indicates Their Role in Calcium Signaling and Plant Defense Against Insect Attack
Source: Front Plant Sci. 2022 Mar 9;13:817950. doi: 10.3389/fpls.2022.817950 (PMC8965522; doi:10.3389/fpls.2022.817950)
Supplement: Supplementary file 1 [file Data_Sheet_1.PDF]

Supplementary Table S1: Comparison of GmCMLs with CMLs of *A. thaliana*

Glycine max CMLs (GmCMLs)	Similar CMLs from *A. thaliana*	Protein similarity
Glyma.10G002200	CML11	85%
Glyma.10G178400	CML11	85%
Glyma.20G211700	CML11	85%
Glyma.01G211700	CML14	84%
Glyma.02G002100	CML8	84%
Glyma.11G030100	CML14	83%
Glyma.11G127500	CML3	81%
Glyma.13G344200	CML3	81%
Glyma.12G052100	CML3	80%
Glyma.19G129800	CML3	78%
Glyma.03G127000	CML3	77%
Glyma.13G074800	CML11	76%
Glyma.03G246800	CML11	73%
Glyma.17G134900	CML14	73%
Glyma.19G244300	CML11	73%
Glyma.03G157800	CML11	70%
Glyma.05G238400	CML25	69%
Glyma.19G160100	CML11	69%
Glyma.20G048900	CML11	68%
Glyma.01G094000	CML25	65%
Glyma.10G161900	CML8	64%
Glyma.07G169100	CML25	63%
Glyma.20G224300	CML8	63%
Glyma.09G067600	CML25	62%
Glyma.07G212000	CML30	60%
Glyma.09G236800	CML42	58%
Glyma.02G143800	CML11	57%
Glyma.04G078400	CML42	57%

Glyma.09G270900	CML42	57%
Glyma.11G217200	CML23	57%
Glyma.18G260700	CML42	57%
Glyma.02G245700	CML23	56%
Glyma.06G079900	CML42	56%
Glyma.18G039500	CML23	56%
Glyma.10G030500	CML11	55%
Glyma.14G215800	CML23	55%
Glyma.16G059300	CML38	51%
Glyma.19G088200	CML38	51%
Glyma.13G159600	CML5	50%
Glyma.17G112000	CML5	50%
Glyma.19G098900	CML11	50%
Glyma.05G245600	CML24	49%
Glyma.06G034700	CML41	49%
Glyma.07G101100	CML24	49%
Glyma.08G053500	CML24	49%
Glyma.16G142500	CML41	49%
Glyma.02G245900	CML25	48%
Glyma.14G170400	CML43	48%
Glyma.14G216000	CML25	48%
Glyma.04G136200	CML41	46%
Glyma.04G136600	CML37	46%
Glyma.04G194800	CML38	46%
Glyma.05G047100	CML38	46%
Glyma.08G093000	CML25	46%
Glyma.17G128900	CML38	46%
Glyma.02G133000	CML25	45%
Glyma.11G157100	CML41	45%
Glyma.11G157200	CML41	45%
Glyma.12G089800	CML8	45%

Glyma.05G138000	CML25	44%
Glyma.11G056500	CML30	44%
Glyma.11G182700	CML8	44%
Glyma.20G227600	CML23	44%
Glyma.01G185600	CML30	43%
Glyma.03G022800	CML25	43%
Glyma.04G136300	CML37	43%
Glyma.17G058200	CML23	43%
Glyma.18G177900	CML41	43%
Glyma.01G144300	CML 25	42%
Glyma.04G144800	CML8	42%
Glyma.05G085200	CML30	42%
Glyma.09G102500	CML37	42%
Glyma.10G160800	CML23	42%
Glyma.12G172000	CML25	42%
Glyma.02G059600	CML39	41%
Glyma.05G015500	CML11	41%
Glyma.14G156300	CML42	41%
Glyma.16G099600	CML8	41%
Glyma.17G175400	CML30	41%
Glyma.12G025200	CML23	40%
Glyma.12G228000	CML23	40%
Glyma.14G085700	CML24	40%
Glyma.18G221300	CML25	40%
Glyma.06G208800	CML8	39%
Glyma.01G076800	CML9	38%
Glyma.04G038300	CML37	38%
Glyma.13G282900	CML12	38%
Glyma.16G022400	CML8	38%
Glyma.19G132800	CML25	38%
Glyma.02G108700	CML3	37%

Glyma.03G130800	CML23	37%
Glyma.04G245000	CML43	37%
Glyma.14G143100	CML24	37%
Glyma.15G055100	CML11	37%
Glyma.20G034200	CML25	37%
Glyma.01G049500	CML3	36%
Glyma.12G197900	CML9	36%
Glyma.13G035100	CML5	36%
Glyma.13G083700	CML25	36%
Glyma.03G259100	CML8	35%
Glyma.07G053300	CML8	35%
Glyma.07G229500	CML25	35%
Glyma.12G103600	CML11	35%
Glyma.19G257800	CML8	35%
Glyma.13G303900	CML9	34%
Glyma.18G101100	CML11	34%
Glyma.11G217300	CML25	33%
Glyma.13G283600	CML8	33%
Glyma.18G100800	CML11	33%
Glyma.02G186900	CML11	32%
Glyma.12G217500	CML8	32%
Glyma.06G255700	CML8	31%
Glyma.08G177600	CML11	31%
Glyma.12G145900	CML12	31%
Glyma.14G113600	CML37	31%
Glyma.17G213600	CML25	30%
Glyma.06G258000	CML11	29%
Glyma.10G287600	CML4	29%
Glyma.14G159700	CML4	29%
Glyma.13G163900	CML10	28%
Glyma.15G006300	CML11	28%

Glyma.07G238700	CML11	27%
Glyma.12G142900	CML8	27%
Glyma.12G217700	CML8	27%
Glyma.13G316100	CML4	27%
Glyma.13G367100	CML11	27%
Glyma.17G107000	CML25	27%
Glyma.08G340400	CML12	26%
Glyma.17G034900	CML11	26%
Glyma.04G064800	CML4	25%
Glyma.06G000700	CML41	25%
Glyma.08G265200	CML12	25%
Glyma.06G066000	CML4	24%
Glyma.12G185400	CML23	24%
Glyma.20G101700	CML7	24%
Glyma.10G106400	CML8	22%
Glyma.17G071900	CML9	22%
Glyma.19G067200	CML12	22%

Supplementary Table S2: List of Primers

		List of Primers	
**Glycine max CML ID**	**CML Name**	**Forward primers**	**Reverse primers**
	EF1b Soy	CTGCCTCTGGGTTGAAGAAG	CCCAAGGTTTCACATCCAAC
**1. Glyma.10G002200**	CML 4	GGTCGATACAGATGGCAATGG	GTGCCTCAACTCACTTGCTG
**2. Glyma.10G178400**	CML 1	AACTTGCCACTGTCATTCGG	GAGCTCCTCCTCTGCATCAG
**3. Glyma.20G211700**	CML 3	GTTCTTGAGCTTGATGGCCA	CATCTGCTCCACCTCCTCAT
**4. Glyma.01G211700**	CML 18	ATCGTGGCTGAAGAGAACCT	GATGTTGGTGAGGATGTGGC
**5. Glyma.02G002100**	CML 2	ACCCCACAGAAGAAGAGCTC	GTGTCTCAACTCACTTGCTGA
**6. Glyma.11G030100**	CML 17	CCCAACTCAAAGCCATCGTG	GATGTTGGTGAGGATGTGGC
**7. Glyma.11G127500**	CML 32	GGAGACGGTTGCATAGACG	CAGCACTGTCCCTAGTTCCT
**8. Glyma.13G344200**	CML 40	GCGACAATGAGGAGGACATG	ATCGACCATGCCATCTCCAT
**9. Glyma.12G052100**	CML 36	GGAGAATCTGGGCATCTTGA	CGGTTCTGATCGAAGACGTT
**10. Glyma.19G129800**	CML 35	GATCAAGGAGAGCTTGCACG	ATTCGCCAAACTCGTCCATG
**11. Glyma.03G127000**	CML 30	GATCAAGGAGAGCTTGCACG	ATTCGCCAAACTCGTCCATG
**12. Glyma.13G074800**	CML 5	GGAACTTGGGACTGTGATGC	CTCCTTAAGCTCCTCCTCGG
**13. Glyma.03G246800**	CML 10	CGGTGGAGGAGTTGCAAATT	CCTCAACTCACTGGGCGATA
**14. Glyma.17G134900**	CML 20	GTCGGATTGCACCATCAGAG	CAACCTTCAAGTGCTTCCCC
**15. Glyma.19G244300**	CML 7	CGGAACCATAGAATTCGGGG	TTCGTCTGTCACCTTCTCCC
**16. Glyma.03G157800**	CML 9	CCAAGACATGATTAGTGAGGTCG	GCTCAGTGGCGGATATGTATC
**17. Glyma.05G238400**	CML 21	GAGCTCAACACCGTCATGC	TCTATCGGTGGTGTCATGGC
**18. Glyma.19G160100**	CML 8	CGATATTGATGGGAATGGGAGT	TCTGCCTCTTCACCTGTCAG
**19. Glyma.20G048900**	CML 6	TGAGCGAGGAACAAGTGTCT	AGGTTCTCTTCAGCCACGAT
**20. Glyma.01G094000**	CML 23	CGGCCACATCAACTTAGGTG	CTTCTGGCACTCGTCAATGG
**21. Glyma.10G161900**	CML 12	GATATGGATGGCAGCGGAAC	CCAGCTCTTCTTCTGTGACC
**22. Glyma.07G169100**	CML 31	CGACGAGGTTTTGGAGAATC	CTGTTCATGTTTCCGGTCA
**23. Glyma.20G224300**	CML 14	TGAAGGAGGTGCTGAGTGAA	GGTTCCACTGCCATTCATATCC
**24. Glyma.09G067600**	CML 38	AAGAGGTTGACGTGGAGGG	ACCATGTTAAGTTCTTCGGCG
**25. Glyma.07G212000**	CML 80	GACTCGGTTCATGAAGTGCC	ATTCTACCGTCGCCATTCCT
**26. Glyma.09G236800**	CML 116	GGCAACTCAACTTCTCCCTTG	TGGTGGCTAAACTCCTCAGG

**27. Glyma.02G143800**	CML 15	ATGGAAATGGCCGGAGTGTA	CGTCTGTTAGCCTCTCTCCC
**28. Glyma.04G078400**	CML 137	GCTGGATGATGAAGTGCCTG	GTCCAACCCCAAGCAATCAA
**29. Glyma.09G270900**	CML 76	GCCGCATCTTCGACATGTTC	CGCCATGAAATCCTCGTACG
**30. Glyma.11G217200**	CML 28	CGAGCTGAAGGAGATGATGG	GAGATCAGCCCGTTCTTGTC
**31. Glyma.18G260700**	CML 117	TGGTTCCAAGGAGCTTTCTGA	GCTGCTTCCTTCAATCCCAA
**32. Glyma.02G245700**	CML 33	GGAAAGGACGACTCGAAGGA	CGTTGCTGATCATTCTCCGG
**33. Glyma.06G079900**	CML 132	GGAGACGGTTGCATAGACG	CAGCACTGTCCCTAGTTCCT
**34. Glyma.18G039500**	CML 27	GCGCAAGATCTTCAGCAAGT	GCCGAACTCCTTCAAGTCAA
**35. Glyma.10G030500**	CML 16	CCGATCATTAGAAGGAAACGCA	GAACGAGTCTTTTAGCTCCTCA
**36. Glyma.14G215800**	CML 29	ACGAGGAGTTGAAACGCATG	GAGATGAGGCCGTTCTTGTC
**37. Glyma.16G059300**	CML 77	GCAGTGACATTGCTCGACAG	AAGACTCCTGGGTGTGATGC
**38. Glyma.19G088200**	CML 56	GGAGGGAAAGAGGAAGAGA	CGAAAGTAAGCACCCCATC
**39. Glyma.13G159600**	CML 25	CTGTTCTCTTCATCGCAGGC	GTCGTTGGTGAAGAGGGAGA
**40. Glyma.17G112000**	CML 22	CTGTTCTCTTCATCGCAGGC	GGTCGTTGGTGAAGAAGAGG
**41. Glyma.19G098900**	CML 11	GAAGGAAGCTTTCAGGGTGT	CAGCCAACATCATCCTCACG

Supplementary Table S3: Tissue-specific expression profile of 31 *GmCMLs* (for 5 *GmCMLS, GmCML11, GmCML18, GmCML25, GmCML38 and GmCML77,* data were not available) (The numbers indicate the RPKM values, retrieved from SoyBase Expression Explorer).

GENE ID	**Flower**	**Young leaf**	**Green pods**	**Root**	**Nodule**	**Stem**	**Seed**
GmCML1	0	6.6	0	4.4	1.1	49.65	0
GmCML2	1	2.5	0.41	4	0.5	0.41	0.5
GmCML3	8.37	2.4	2.79	4.8	10.8	11.16	1.2
GmCML4	5.61	5.59	5.61	14	0	39.31	1.39
GmCML5	131.55	194	306.95	155.19	96.99	131.55	96.99
GmCML6	128.55	305	205.68	274.5	213.5	102.84	91.5
GmCML7	0.6	0.2	0	0	0	0.6	0.2
GmCML8	0.74	1.4	0	1	0.2	0.74	0.4
GmCML9	0	0.69	1.38	7	0.69	0	0
GmCML10	0.98	2.4	0.98	0.8	0.8	0.32	0
GmCML12	1.39	8.39	0	0	0	1.39	0
GmCML14	0.45	1.6	0	0.4	0.8	0	0.4
GmCML15	0	0	0	0	0	0	0
GmCML16	0	0	0	0	0	0	0
GmCML17	24.13	44.8	96.54	57.6	6.4	48.27	32

GmCML20	1.48	0	0	0	0	0	0
GmCML21	4.23	0.1	0	0	0	0.7	0
GmCML22	7.1	0	0	0	0	14.2	0
GmCML23	0	0	0	0	0	0	0
GmCML28	75.21	39.89	37.6	5.69	0	125.35	0
GmCML29	13.57	6.49	6.78	10.39	6.49	18.09	13
GmCML30	6.81	1	0.68	1	0	0.68	2
GmCML32	3.14	2	3.14	20	6	0	2
GmCML33	19.76	12	7.9	7.2	8.4	13.83	10.8
GmCML35	4.15	0	0.41	0	0	0.83	0
GmCML40	76.47	55.5	254.92	111	111	101.96	0
GmCML76	27.02	0.69	5.4	5.6	0.69	5.4	0.69
GmCML80	52.48	4.59	5.24	6.89	2.29	20.99	0
GmCML116	2.62	0.5	1.31	5	2	13.13	0
GmCML117	2.98	0.8	2.98	8	5.6	10.45	0.8
GmCML137	8.37	0	0	11.39	38	8.37	0

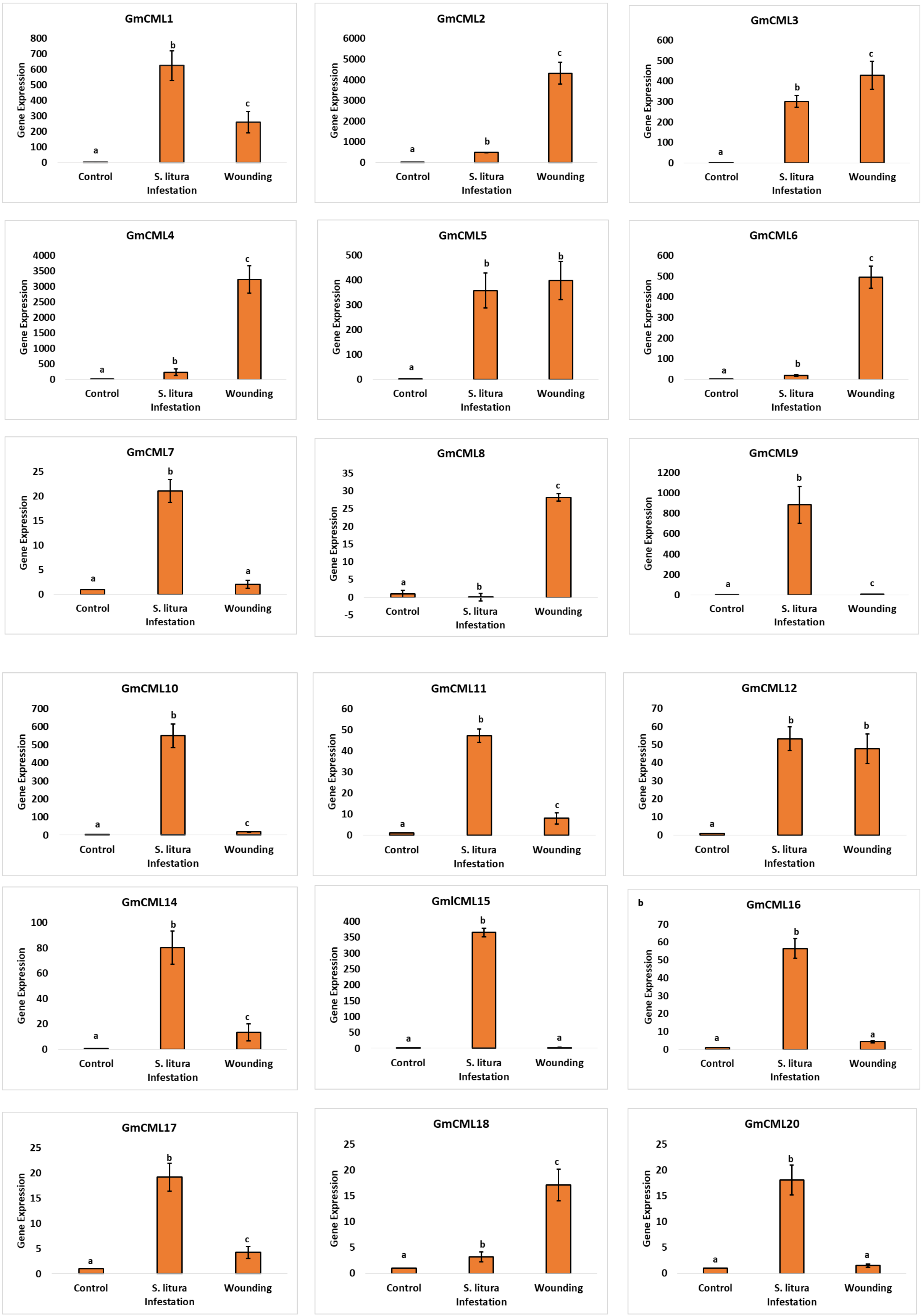
GmCML1
GmCML2
GmCML3
GmCML4
GmCML5
GmCML6
GmCML7
GmCML8
GmCML9
GmCML10
GmCML11
GmCML12
GmCML14
GmlCML15
GmCML16
GmCML17
GmCML18
GmCML20
Gene Expression
Control
S. litura Infestation
Wounding

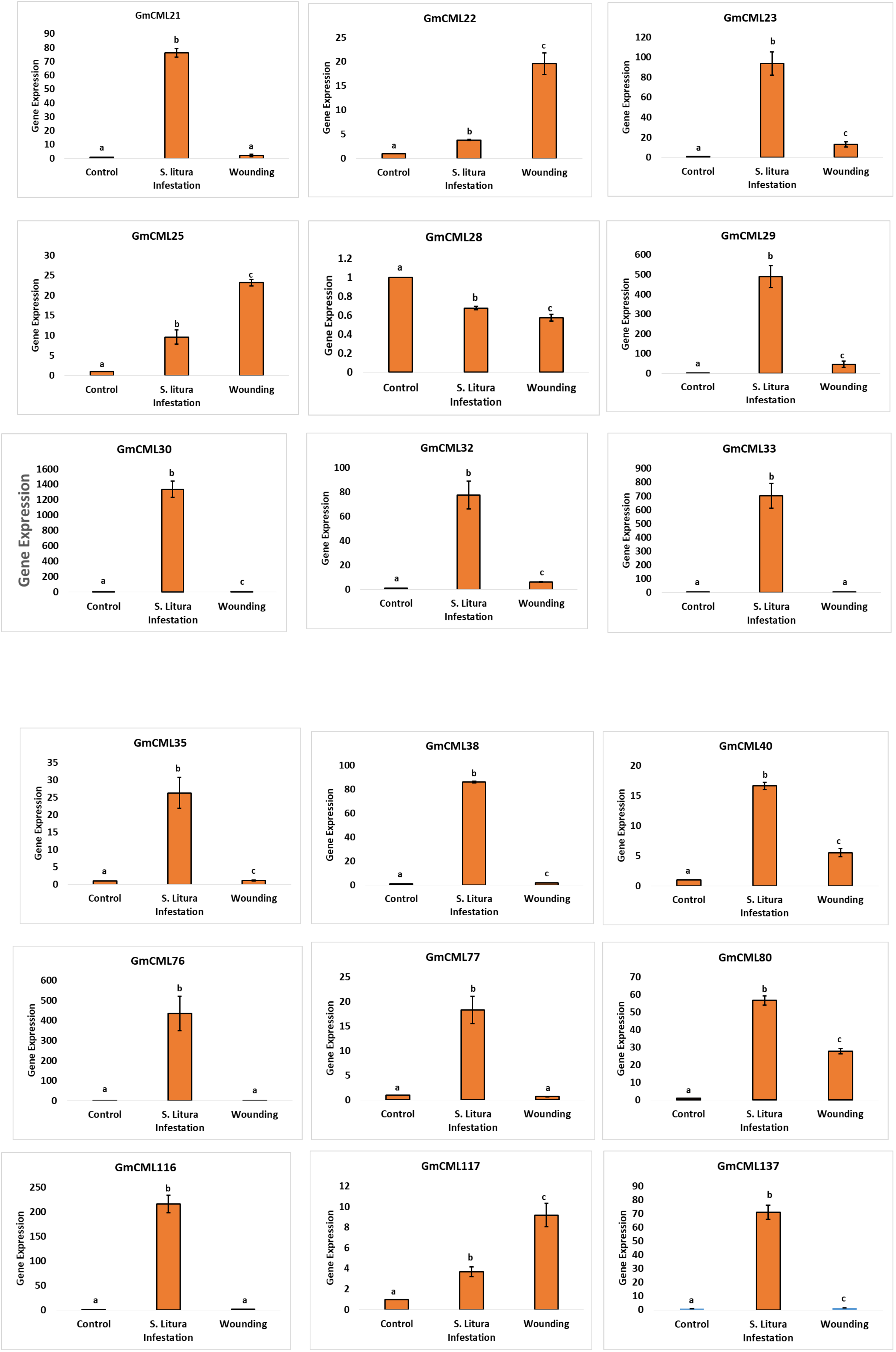
GmCML21
Gene Expression
90 80 70 60 50 40 30 20 10 0
a b a
Control
S. litura Infestation
Wounding
GmCML22
Gene Expression
25 20 15 10 5 0
a b c
Control
S. litura Infestation
Wounding
GmCML23
Gene Expression
120 100 80 60 40 20 0
a b c
Control
S. litura Infestation
Wounding
GmCML25
Gene Expression
30 25 20 15 10 5 0
a b c
Control
S. litura Infestation
Wounding
GmCML28
Gene Expression
1.2 1 0.8 0.6 0.4 0.2 0
a b c
Control
S. Litura Infestation
Wounding
GmCML29
Gene Expression
600 500 400 300 200 100 0
a b c
Control
S. Litura Infestation
Wounding
GmCML30
Gene Expression
1600 1400 1200 1000 800 600 400 200 0
a b c
Control
S. Litura Infestation
Wounding
GmCML32
Gene Expression
100 80 60 40 20 0
a b c
Control
S. Litura Infestation
Wounding
GmCML33
Gene Expression
900 800 700 600 500 400 300 200 100 0
a b a
Control
S. Litura Infestation
Wounding
GmCML35
Gene Expression
35 30 25 20 15 10 5 0
a b c
Control
S. Litura Infestation
Wounding
GmCML38
Gene Expression
100 80 60 40 20 0
a b c
Control
S. Litura Infestation
Wounding
GmCML40
Gene Expression
20 15 10 5 0
a b c
Control
S. Litura Infestation
Wounding
GmCML76
Gene Expression
600 500 400 300 200 100 0
a b a
Control
S. Litura Infestation
Wounding
GmCML77
Gene Expression
25 20 15 10 5 0
a b a
Control
S. Litura Infestation
Wounding
GmCML80
Gene Expression
70 60 50 40 30 20 10 0
a b c
Control
S. Litura Infestation
Wounding
GmCML116
Gene Expression
250 200 150 100 50 0
a b a
Control
S. Litura Infestation
Wounding
GmCML117
Gene Expression
12 10 8 6 4 2 0
a b c
Control
S. Litura Infestation
Wounding
GmCML137
Gene Expression
90 80 70 60 50 40 30 20 10 0
a b c
Control
S. Litura Infestation
Wounding

Supplementary Figure S1: The graphical representation of expression profiles of *GmCMLs* upon insect-infestation and wounding. Data is represented as histograms using fold change values of expression data obtained from qPCR of *GmCML*s. The qPCR data were analyzed utilizing the $2^{-\Delta\Delta CT}$ method and statistical analysis was performed using ANOVA and Tukey's test (P <0.05).

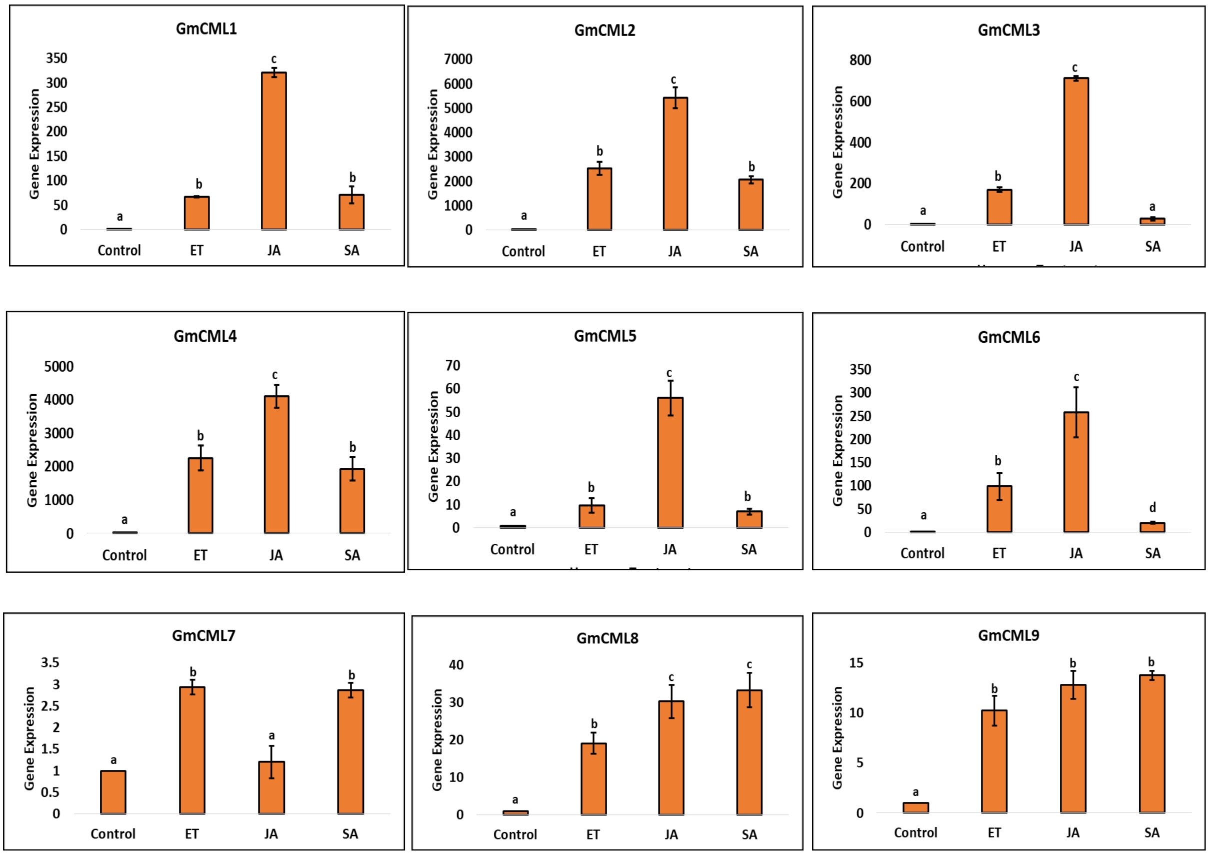

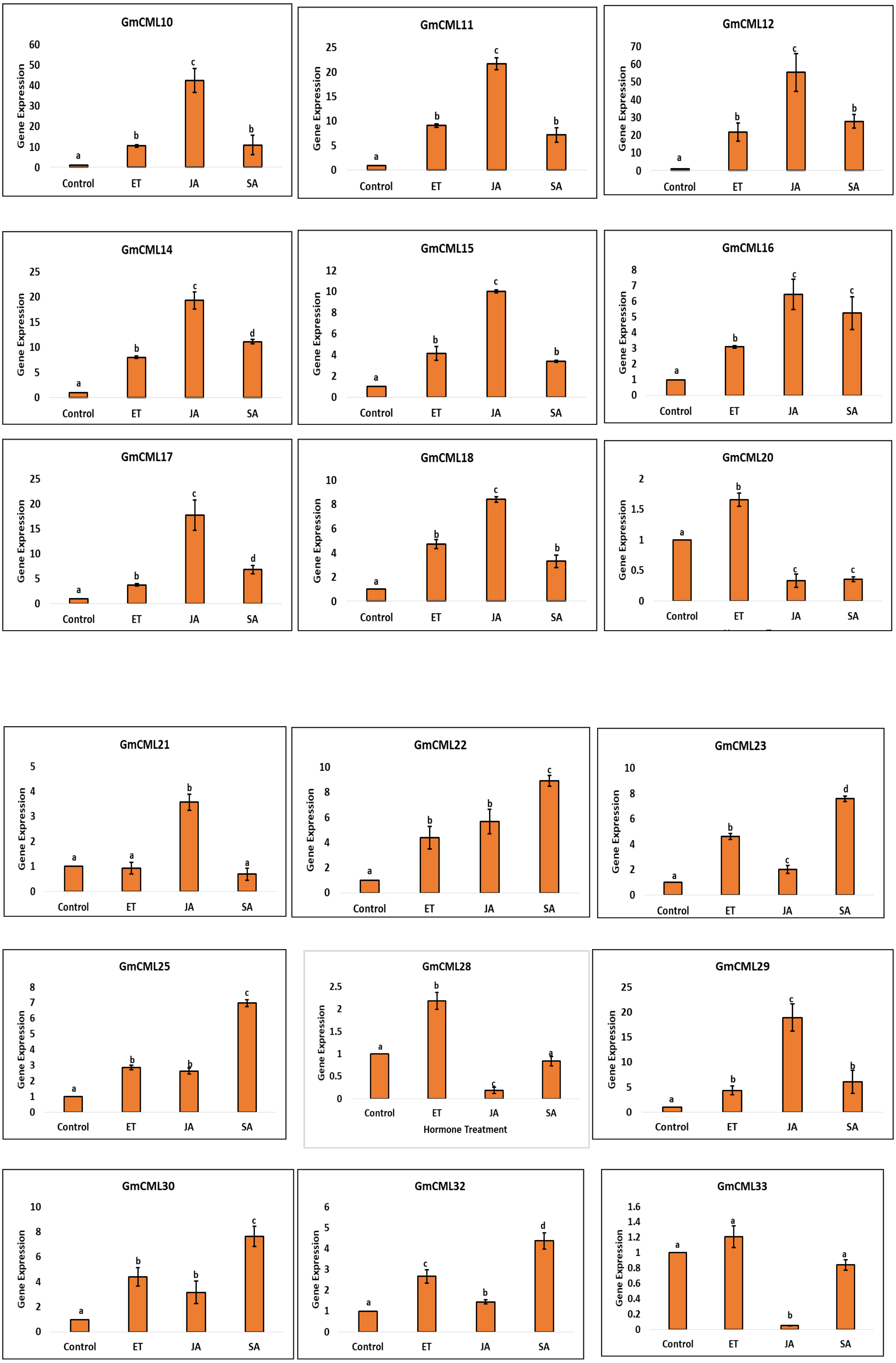
GmCML10
Gene Expression
Control
ET
JA
SA
GmCML11
Gene Expression
Control
ET
JA
SA
GmCML12
Gene Expression
Control
ET
JA
SA
GmCML14
Gene Expression
Control
ET
JA
SA
GmCML15
Gene Expression
Control
ET
JA
SA
GmCML16
Gene Expression
Control
ET
JA
SA
GmCML17
Gene Expression
Control
ET
JA
SA
GmCML18
Gene Expression
Control
ET
JA
SA
GmCML20
Gene Expression
Control
ET
JA
SA
GmCML21
Gene Expression
Control
ET
JA
SA
GmCML22
Gene Expression
Control
ET
JA
SA
GmCML23
Gene Expression
Control
ET
JA
SA
GmCML25
Gene Expression
Control
ET
JA
SA
GmCML28
Gene Expression
Control
ET
JA
SA
Hormone Treatment
GmCML29
Gene Expression
Control
ET
JA
SA
GmCML30
Gene Expression
Control
ET
JA
SA
GmCML32
Gene Expression
Control
ET
JA
SA
GmCML33
Gene Expression
Control
ET
JA
SA

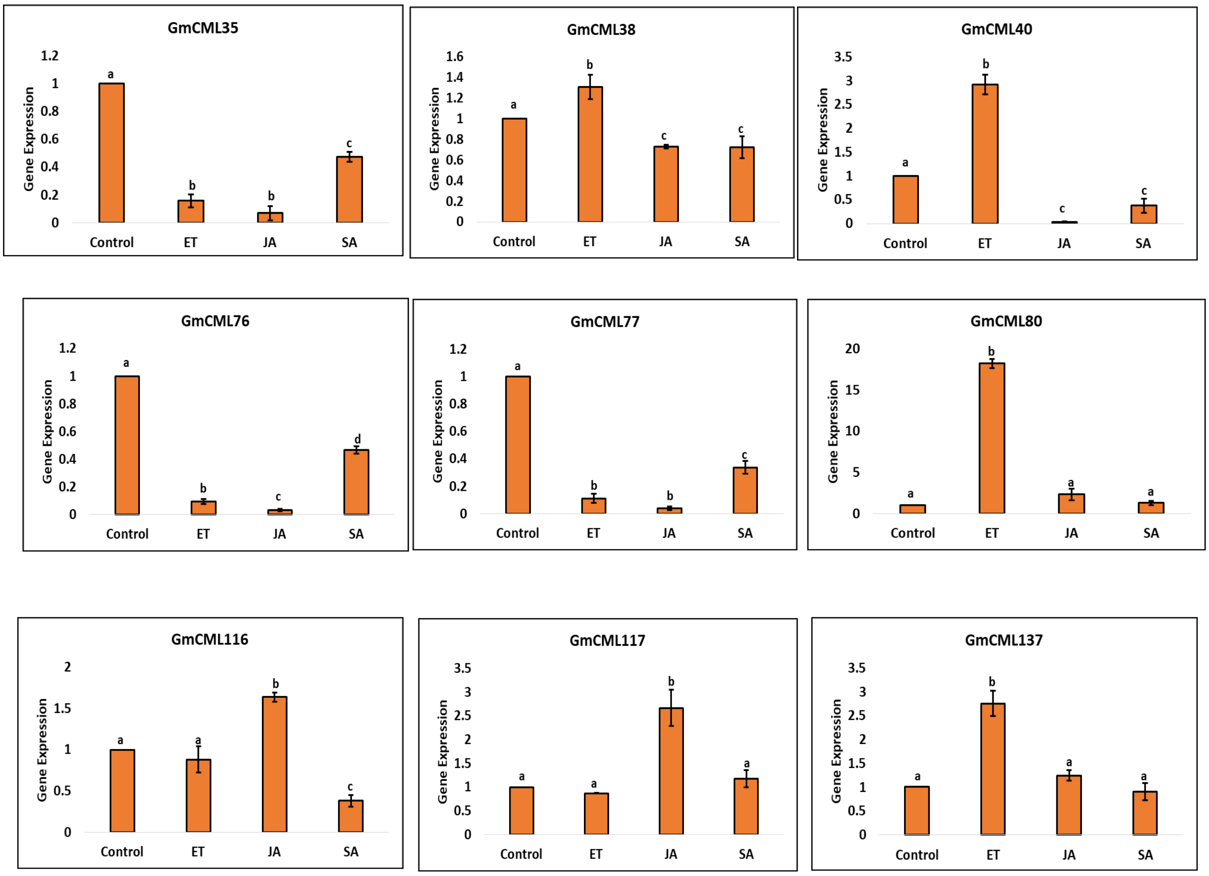

Supplementary Figure S2: The graphical representation of expression profiles of *GmCMLs* on application of JA, SA and ET. Data is represented as histograms using fold change values of expression data obtained from qPCR of *GmCML*s. The qPCR data were analyzed utilizing the $2^{-\Delta\Delta CT}$ method and statistical analysis was performed using ANOVA and Tukey's test ($P < 0.05$).
